# An Integrate-and-Fire Spiking Neural Network Model Simulating Artificially Induced Cortical Plasticity

**DOI:** 10.1523/ENEURO.0333-20.2021

**Published:** 2021-03-11

**Authors:** Larry Shupe, Eberhard Fetz

**Affiliations:** 1 Department of Physiology and Biophysics, University of Washington, Seattle, WA 98195; 2 Department of Physiology and Biophysics and Regional Primate Research Center, University of Washington, Seattle, WA 98195

**Keywords:** brain-machine interface, cerebral cortex, neural network, plasticity, spiking

## Abstract

We describe an integrate-and-fire (IF) spiking neural network that incorporates spike-timing-dependent plasticity (STDP) and simulates the experimental outcomes of four different conditioning protocols that produce cortical plasticity. The original conditioning experiments were performed in freely moving non-human primates (NHPs) with an autonomous head-fixed bidirectional brain-computer interface (BCI). Three protocols involved closed-loop stimulation triggered from (1) spike activity of single cortical neurons, (2) electromyographic (EMG) activity from forearm muscles, and (3) cycles of spontaneous cortical beta activity. A fourth protocol involved open-loop delivery of pairs of stimuli at neighboring cortical sites. The IF network that replicates the experimental results consists of 360 units with simulated membrane potentials produced by synaptic inputs and triggering a spike when reaching threshold. The 240 cortical units produce either excitatory or inhibitory postsynaptic potentials (PSPs) in their target units. In addition to the experimentally observed conditioning effects, the model also allows computation of underlying network behavior not originally documented. Furthermore, the model makes predictions about outcomes from protocols not yet investigated, including spike-triggered inhibition, γ-triggered stimulation and disynaptic conditioning. The success of the simulations suggests that a simple voltage-based IF model incorporating STDP can capture the essential mechanisms mediating targeted plasticity with closed-loop stimulation.

## Significance Statement

Previous experiments have shown that the synaptic connections between neurons in cerebral cortex can be modified by different activity-dependent stimulation methods. Understanding the mechanisms that mediate these changes is key to designing effective protocols for producing targeted plasticity in clinical and basic science applications. We describe a simple but powerful neural network model that replicates a wide range of experimental results on targeted cortical plasticity. The model consists of integrate-and-fire (IF) spiking units and incorporates spike-timing-dependent plasticity (STDP). It provides important insights into the mechanisms underlying induction of plasticity with bidirectional brain-computer interfaces (BCIs). The model also predicts the outcomes of several novel and untested conditioning protocols.

## Introduction

Computational neural network models provide a powerful tool for understanding mechanisms of neural computation and for exploring network behavior in ways that physiological recordings cannot (Fetz, 1993; Fetz and Shupe, 1995; Gerstner and Kistler, 2002; Gerstner et al., 2014). Neural networks consisting of integrate-and-fire (IF) spiking units have proven useful in studying network dynamics produced by spiking neurons (for reviews, see Jolivet et al., 2004; Burkitt, 2006; Gilson et al., 2009a,b,c,d; Ponulak and Kasinski, 2011). The IF units typically sum inputs to produce a simulated membrane potential function that triggers a spike when it reaches a threshold. The inputs can simulate postsynaptic potentials (PSPs) with rise time and decay; the units can also incorporate biophysical conductances (Izhikevich, 2003). Spike-timing-dependent plasticity (STDP) rules (Bi and Poo, 2001; Caporale and Dan, 2008; Feldman, 2012; Markram et al., 2012) can be incorporated into IF networks to investigate consequent changes in synaptic connections on network dynamics. For example, large networks of biophysically realistic spiking units with STDP have been shown to form functional interacting groups (Izhikevich et al., 2004; Koene and Hasselmo, 2005; Litwin-Kumar and Doiron, 2014; Bono and Clopath, 2017) and capture global changes induced by conditioning (Song et al., 2013). We here used an IF network with STDP to simulate experimental results from recent physiological conditioning studies performed with a closed-loop brain-computer interface (CL-BCI).

The strength of synaptic connections between motor cortical neurons has been experimentally modified by several different conditioning protocols. In non-human primates (NHPs), CL-BCIs have induced plasticity with spike-triggered stimulation of neighboring cortical sites during free behavior (Jackson et al., 2006). After a day of conditioning, the output effects on muscles and isometric wrist responses evoked by microstimulation of the recording site (labeled A) included the output effects evoked from the stimulation site (B), consistent with a strengthening of connections from A to B. Changes occurred only for spike-stimulus delays of 50 ms or less, consistent with the time course of STDP. These changes lasted for up to 10 d postconditioning. A second conditioning protocol triggered cortical stimulation from pulses generated by electromyographic (EMG) activity of a forearm muscle (Lucas and Fetz, 2013). This produced changes in the output effects evoked by stimulating the cortical site (A) that was associated with the recorded muscle to now include effects evoked from the stimulated site (B). Again, the results were consistent with a strengthening of connections from A to B. A third conditioning protocol used paired sequential stimulation of sites A and B and found changes in the magnitude of potentials at B evoked by stimulation at A (Seeman et al., 2017). These effects were found at some, but not all site pairs, and were again seen for stimulus intervals of 30 ms or less. A fourth conditioning protocol produced cortical plasticity using a CL-BCI to stimulate site A during specific phases of spontaneous β oscillations at B (Zanos et al., 2018). This produced transient changes in the connection from A to B; the connections increased or decreased, depending on whether stimuli were delivered during the phase in which neurons at B would tend to be depolarized or hyperpolarized, respectively. These changes in connectivity were induced by spontaneous oscillatory episodes with three or more cycles and decayed within seconds.

We here investigated whether the neural mechanisms underlying these four conditioning protocols could be captured by an IF neural network model that incorporates STDP. This approach differs from a previous neural network model using populations of Poisson firing units to analytically compute the net effects produced by spike-triggered stimulation (Lajoie et al., 2017; see Discussion). Our IF model replicated the results of all four conditioning protocols with a single set of network parameters and enabled derivations beyond the original experimental observations, providing a more complete picture of conditioned changes and insights into the effects of relevant network parameters. Furthermore, the model also provided totally novel predictions about the outcomes of possible conditioning experiments that have not yet been performed. Thus, there is a productive symbiotic relation between the model and physiological experiments. Our IF model provides a powerful tool for elucidating the synaptic mechanisms underlying cortical plasticity and discovering new conditioning protocols.

## Materials and Methods

### IF network model

**Figure 1.**
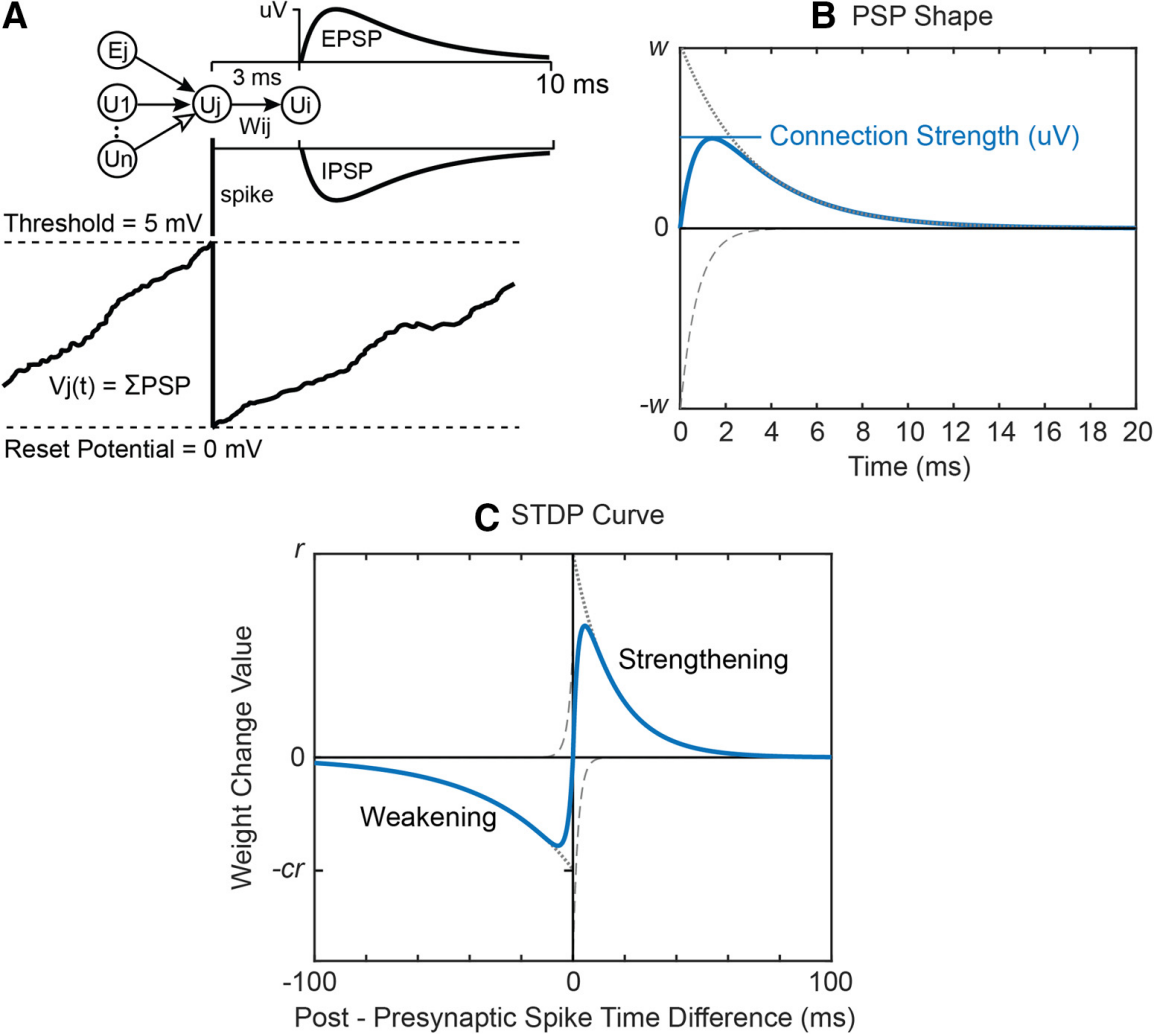
IF model. ***A***, External input (Ej) and spiking input from connected units (U1 to Un) are summed into a target unit’s potential (Vj). When a unit’s potential reaches threshold it is reset to 0 and the unit sends a spike to all its target units. The spike evokes an excitatory or inhibitory PSP proportional to synaptic weight *w_ij_*. ***B***, PSP Shape is calculated as the difference between two exponential functions (dotted and dashed lines). ***C***, The STDP curve shows how much weight *w_ij_*is changed given the difference in spike times between the target unit i and the source unit j.

The IF network model consists of 360 IF units. Each unit maintains a potential *V_i_(t)* which represents the sum of the synaptic inputs from units with connections to unit *i* (Fig. 1*A*). *V_i_(t)* is calculated at discrete time steps (h) of 0.1 ms. When *V_i_(t)* exceeds a threshold θ, the unit “fires” and its spike function *U_i_(t)* is set to 1 for that time step (Eq. 3). Each time a unit fires, its potential is reset to zero on the next time step and an output spike is initiated from that source unit to all its target units.

The responses of the modeled units to spiking inputs represent the PSPs of physiological neurons (Fig. 1*B*). The form of the PSP is calculated as the difference between two exponential decay functions as follows:

(1) PSP(t)=w(e−(t−tspike)/τs−e−(t−tspike)/τf), if t≥tspike;0 otherwise.

The time constants *τ_s_* (slower decay) and *τ_f_* were chosen to give reasonably shaped PSPs, rising rapidly from zero to a maximum, and then decaying more slowly back toward zero (Fig. 1*B*). The parameter w is the connection weight; this parameter is modified by the plasticity calculations. A related parameter, the connection strength is measured by the PSP maximum, which represents the synaptic efficacy in voltage change, relatable to distance to threshold. The relation between the weight and strength depends on *τ_s_* and *τ_f_* (Fig. 1*B*). The calculation of *V_i_(t)* is greatly simplified by using the same value of *τ_s_* and *τ_f_* for all connections. This allows *V_i_(t)* to be represented by two leaky integrators *V^s^_i_(t)* and *V^f^_i_(t)* that decay with these constants (Eqs. 4, 5); they both receive the same spiking input and reset with the same spike time function *U_i_(t)*. Here, the Euler method is used to estimate the exponential decay functions. We let *A_i_(t)* be the sum of all incoming spike activity to unit *i* at time *t* (Eq. 6), and then calculate the unit potential *V_i_(t)* and spike time function *U_i_(t)* as follows:

(2) $$V_{i}\left(t\right)={\text{V}}_{i}^{s}\left(t\right)-{\text{V}}_{i}^{f}\left(t\right)$$

(3) $$U_{i}\left(t\right)=1,if\;V_{i}\left(t\right)>\theta ;0\;otherwise$$

(4) $${\text{V}}_{i}^{s}\left(t+h\right)=(1–h/\tau_{s}){\text{V}}_{i}^{s}\left(t\right)+A_{i}\left(t\right),if\;U_{i}\left(t\right)=0;0\;if\;U_{i}\left(t\right)=1$$

(5) $${\text{V}}_{i}^{f}\left(t+h\right)=(1–h/\tau_{f}){\text{V}}_{i}^{f}\left(t\right)+A_{i}\left(t\right),if\;U_{i}\left(t\right)=0;0\;if\;U_{i}\left(t\right)=1.$$

Each connection linking source unit *j* to target unit *i* at time *t* is defined by a weight *w_ij_(t).* The weight is positive or negative for excitatory or inhibitory units, respectively. Non-existent connections assume a weight of 0. Axonal plus dendritic conduction times are modeled as a global delay parameter *d* for all connections. To provide additional background activity each unit also receives external input *E_i_(t)* the sum of all external spiking activity arriving at unit *i* at time *t* (Eq. 7). *A_i_(t)* is defined as follows:

(6) $$A_{i}\left(t\right)=E_{i}\left(t\right)+\overset{n}{\underset{j=1}{\sum }}\left(U_{j}\left(t-d\right)\cdot w_{ij}(t)\right),$$

where n equals the total number of units. The units are subdivided into three populations called “columns,” representing recorded, stimulated and control sites (see Network topology, below). To provide background spontaneous activity, each column receives excitatory external inputs *E_i_(t)* that are a combination of correlated inputs *E^c^_i_(t)* and uncorrelated inputs *E^u^_i_(t)*:

(7) $$E_{i}\left(t\right)=E_{i}^{c}\left(t\right)+E_{i}^{u}\left(t\right).$$

These inputs produce PSPs with the same waveform as other connections in the network, varying only in amplitude. For each column, the correlated events occur with a given probability at each time step; every unit in the column receives the correlated input at a random time within a Gaussian distribution with a SD of 3 ms. Uncorrelated events occur with a separate independent probability at every time step for each unit. The delivery probabilities of correlated and uncorrelated events can be modulated in time to simulate different dynamics of background activity (e.g., oscillations). For our networks, *E^c^_i_(t)* and *E^u^_i_(t)* assume a fixed connection weight at times of external spike delivery and are 0 otherwise.

### Plasticity rule

When STDP is active, connection weights are modified based on the relative firing times of the target and source units. The STDP curve (Fig. 1*C*) shows how much weight *w_ij_* changes as a function of the difference in spike times between target unit *i* and source unit *j*. The curve has two components: a strengthening portion for positive differences *Δt* between time of postsynaptic spike and arrival of presynaptic spike that will strengthen the weight, and a weakening portion for negative time differences that will weaken the weight. Each half of the STDP function was approximated with exponential decay functions similar to the PSP function except with longer time constants ($a_{s},a_{f},b_{s},b_{f}$) and with amplitudes controlled by the training factor r and a weakening factor *c*:

(8) $$STDP(\Delta t)=\{\begin{array}{c} r\left(e^{-\Delta t/a_{s}}-e^{-\Delta t/a_{f}}\right),\Delta t\geq 0\\ -cr\left(e^{\Delta t/b_{s}}-e^{\Delta t/b_{f}}\right),\Delta t<0 \end{array}.$$

The weakening side of the STDP curve has a lower amplitude but decays more slowly than the strengthening side, and has larger overall area (Caporale and Dan, 2008). The same STDP function was used for excitatory and inhibitory synaptic connections, for which there is empirical support (Haas et al., 2006). For further discussion of this and alternate STDP functions for inhibitory synapses, see Discussion.

Choosing the right amount of weakening versus strengthening is important to the evolution of the network connections. If the weakening factor is too small, the greater area of the strengthening side of the STDP curve will eventually cause all weights to grow to a maximum limit. If the weakening factor is too high, the greater area of the weakening side will push all weights strongly toward zero. We chose a weakening factor of *c* = 0.55, so that the network sustained low weight values in the absence of any conditioning stimuli but showed noticeable increases in some weights when conditioning was applied.

For a single weight *w_ij_* and time difference between spikes of unit *i* and arriving spikes of unit *j*, the change in *w_ij_* is equal to the value taken from the STDP function in Figure 1*C*. To facilitate computation of weight changes, strengthening and weakening potentials are maintained for each unit similar to the way unit potential *V_i_(t)* sums PSPs (except with no threshold crossing reset). *S_j_(t)* sums the strengthening side of the STDP curve for each source unit *j* using time constants *a_s_* and *a_f_* with input activity *U_j_(t–d)*. *T_i_(t)* sums the weakening side for each target unit i using time constants $b_{s}$ and $b_{f}$ with input activity *U_i_(t)*. Thus, when a spike occurs, weight changes can be calculated with respect to previous spikes by taking signed and scaled values of *S_j_(t)* and *T_i_(t)* (Eq. 15).

(9) $$S_{j}\left(t\right)=S_{j}^{s}\left(t\right)-S_{j}^{f}\left(t\right)$$

(10) $$S_{j}^{s}\left(t+h\right)=(1–h/a_{s})S_{j}^{s}\left(t\right)+U_{j}\left(t-d\right)$$

(11) $$S_{j}^{f}\left(t+h\right)=(1–h/a_{f})S_{j}^{f}\left(t\right)+U_{j}\left(t-d\right)$$

(12) $$T_{i}\left(t\right)=T_{i}^{s}\left(t\right)-T_{i}^{f}\left(t\right)$$

(13) $$T_{i}^{s}\left(t+h\right)=(1–h/b_{s})T_{i}^{s}\left(t\right)+U_{i}\left(t\right)$$

(14) $$T_{i}^{f}\left(t+h\right)=(1–h/b_{f})T_{i}^{f}\left(t\right)+U_{i}\left(t\right).$$

The change to *w_ij_* at a given time step is:

(15) $$\Delta w_{ij}\left(t\right)=r\;\text{sgn}\left(w_{ij}\left(t\right)\right)\left(S_{j}\left(t\right)U_{i}\left(t\right)-cT_{i}\left(t\right)U_{j}\left(t-d\right)\right)$$

(16) $$w_{ij}\left(\text{t}+h\right)=F\left(w_{ij}\left(\text{t}\right)+\Delta w_{ij}\left(t\right)\right).$$

The function *F(w)* clips excitatory weights to the range *w_min_* ≤ *w* ≤ *w_max_*, and inhibitory weights to the range -*w_max_* ≤ *w* ≤ -*w_min_*. This function can be modified to squash weight changes as the weight approaches min and max limits, but for the simulations here no graded squashing was performed. The standard parameter values used in simulations are shown in Table 1.

**Table 1:** Parameter values

Number of excitatory units in each column	40
Number of inhibitory units in each column	40
Unit firing threshold	5 mV
Conduction delay between cortical units	3 ms
Conduction delay to motor outputs	10 ms
External Input PSP strength	350 μV
Unit potential time constants *τ_s_*, *τ_f_*	3.2, 0.8 ms
External input rate	1800 spikes/s
External input correlated spike percentage	30%
External input correlated spike SD	3 ms
Training factor r	100
Weakening factor c	0.55
STDP strengthening curve time constants *a_s_*, *a_f_*	15.4, 2 ms
STDP weakening curve time constants *b_s_*, *b_f_*	33.3, 2 ms
Conditioning stimulus intensity	2 mV
Maximum connection strength (used to calculate w_max_)	500 μV
Minimum weight *w_min_*	1
Weight change squashing (weight dependence)	0 (none)
Probability of excitatory cortical connections	1/6
Probability of inhibitory connections	1/3
Probability of corticomotoneuronal connections	1/3
Number of motoneurons	40
Motoneuron thresholds	5–6 mV
Motor unit potential size	0.5–1.5 mV
Available simulation time step sizes h	0.1 (default), 0.05, 0.025, 0.02, 0.01 ms

### Network topology

**Figure 2.**
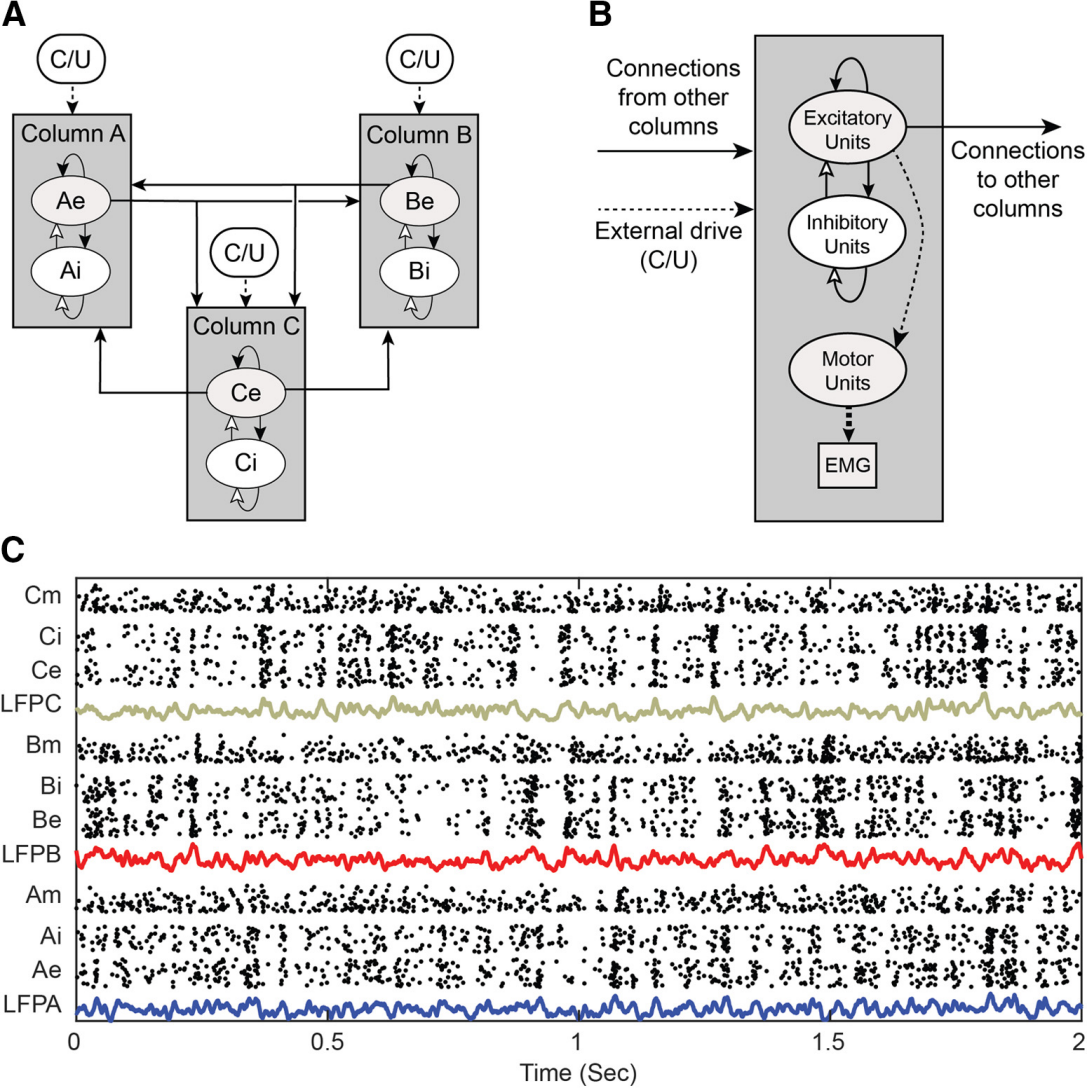
Network connectivity. ***A***, Cortical network configuration. Units are grouped into three columns A–C. Each column contains 40 excitatory units (e.g., Ae1 … Ae40) and 40 local inhibitory units (Ai) all sparsely interconnected. Only excitatory units project to other columns. Columns also receive external input, consisting of correlated and uncorrelated (C/U) exponentially distributed spikes. ***B***, Column configuration. Associated with each cortical column is a pool of motor units that produce muscle potentials (EMG). The dashed lines designate unmodifiable connections and solid lines represent modifiable connections. ***C***, Example spiking activity of excitatory (e), inhibitory (i), and motor units (m) associated with each column. LFP is the sum of within-column PSPs in e and i units.

The network contains 240 cortical units grouped into three columns, A, B, and C, as shown in Figure 2*A*. Each column has 40 excitatory units (e) that project only positive weights, and 40 inhibitory units (i) that project only negative weights. Excitatory units connect sparsely to all other units in the network and inhibitory units connect less sparsely to all units within the same column. The probability of each possible connection is listed in Table 1. Unit self-connections are not allowed. Each column also has a simulated local field potential (LFP) which is the sum of all PSPs occurring within a column.

To provide background spontaneous activity each column receives an external excitatory bias input generated separately for each column. Some bias inputs provide correlated spikes to each unit in a column, and others provide independent uncorrelated spikes to all units (Fig. 2*A, C*/*U*), for a combined mean bias rate of 1800 spikes/s to each unit. The ratio of the number of correlated to uncorrelated spikes each unit receives can be modified to control the degree of synchronization within a column. The mean bias rate can also be modulated over time, for example to generate oscillatory activity or simulate behavioral activation.

To simulate experiments that involved recording muscle activity, the network includes pools of motor units driven by the cortical columns (Fig. 2*B*). Associated with each column is a pool of 40 motoneurons that receive inputs from a third of the excitatory units of that column, as well as from uncorrelated external drive to simulate all additional inputs. Consistent with the size principle (Henneman et al., 1965), the pool included a range of small to large motoneurons, with increasing thresholds from 5 to 6 mV and increasing muscle unit potentials from 0.5 to 1.5 mV. Multiunit EMG activity was simulated by summing all the muscle unit potentials in the same manner as for unit PSPs and then filtering the result with a 100- to 2500-Hz bandpass filter.

Figure 2*C* shows the simulated spiking activity of all units under steady-state conditions. Also plotted are the corresponding LFPs for each column. The coordinated bursts of activity are produced by 30% correlated external bias drive.

### Conditioning stimulation

The effect of electrical stimulation on a stimulated unit is modeled by a large and immediate deflection of its potential toward threshold, computed by adding a large stimulus pulse to the unit’s *V^s^_i_(t),* proportional to stimulus intensity. The standard conditioning stimulus produces a step in *V_i_*(t) of amplitude 2 mV. The standard testing stimulus for evoking potentials produces a step in *V_i_*(t) of amplitude 3 mV. Normally a conditioning stimulus is applied to all units in a column simultaneously, causing many of them to fire. This burst of spikes will evoke a measurable response in the LFP of other columns, called the evoked potential (EP). The EP is a measure of the net synaptic strengths from the stimulated column to the recorded column. The size of each EP is calculated as the difference between the amplitude of the LFP peak after the stimulus compared with prestimulation baseline LFP. The average change in the EP amplitude produced by conditioning is quantified as the percent increase of the average EP amplitude after conditioning compared with before conditioning:

(17) $$EP\;Increase\%=\frac{100(Post\;Conditioned\;Average\;EP–Preconditioned\;Average\;EP)}{Preconditioned\;Average\;EP}.$$

### Implementation

The network has many modifiable parameters that can affect the outcome of a simulation (Table 1). Temporal parameters are generally scaled in milliseconds. The network runs in time steps of 0.1 ms to strike a balance between accuracy and computation time, although other values of h are available in the code provided online. Connection strengths, stimulation amplitudes, and PSP-related parameters are scaled in microvolts to relate such values to physiological variables. Connection strengths are initially small values (20–60% of maximum) taken randomly from a uniform distribution.

Most networks were run in 10-s time blocks for 2000 s of simulated time, divided into four periods of 500 s each (Table 2). During the preconditioning period STDP is on and the network settles into an unconditioned steady state. During the two testing stages before and after conditioning the STDP calculations are turned off, allowing graphs and tests to be compiled while the network runs with static weights. The conditioning protocol is run during the conditioning period with STDP on.

**Table 2:** Sequential training periods

Period	Preconditioning	Preconditioning testing	Conditioning	Postconditioning testing
Plasticity	On	Off	On	Off
Conditioning	Off	Off	On	Off

### Tetanic stimulation

To control for the effects of stimulation alone, tetanic stimulation was performed with a 10-Hz Poisson spike train with an imposed 10-ms refractory period. This can have a strengthening effect on connections from the stimulated column to the other two columns, and a weakening effect in the reciprocal directions (see “Control for tetanic stimulation”, below). These effects were minimized by selecting a weakening factor large enough to yield preconditioned networks with small weights that would readily show the effects of conditioned strengthening. There is substantial variation in the sizes of conditioned EPs because of differences in initial conditions, the randomness of the external input, and the magnitude of the training factor parameter. However, averaging over a set of simulations using 10 different initial conditions yields a reliable measure of the effects of tetanic stimulation. These tetanic effects can support or oppose other conditioning. For example, any protocol to strengthen A→B connections that employs stimulation on column B must overcome the tendency for tetanic stimulation on B to weaken those connections. On the other hand, protocols that stimulate column A (like cycle-triggered stimulation) should be evaluated relative to simple tetanic stimulation of column A.

### Code accessibility

The MATLAB (v. 2019a) code for the IF network model is available online at https://github.com/lshupe/spikenet. Online material includes documentation and instructions on running the model under Windows 10, as well as further explanation of effects of network parameters. The code includes options not exercised in these simulations, such as including a squashing function for graded weight changes and running with finer temporal resolution (which did not significantly alter the main results).

## Results

### Spike-triggered stimulation

**Figure 3.**
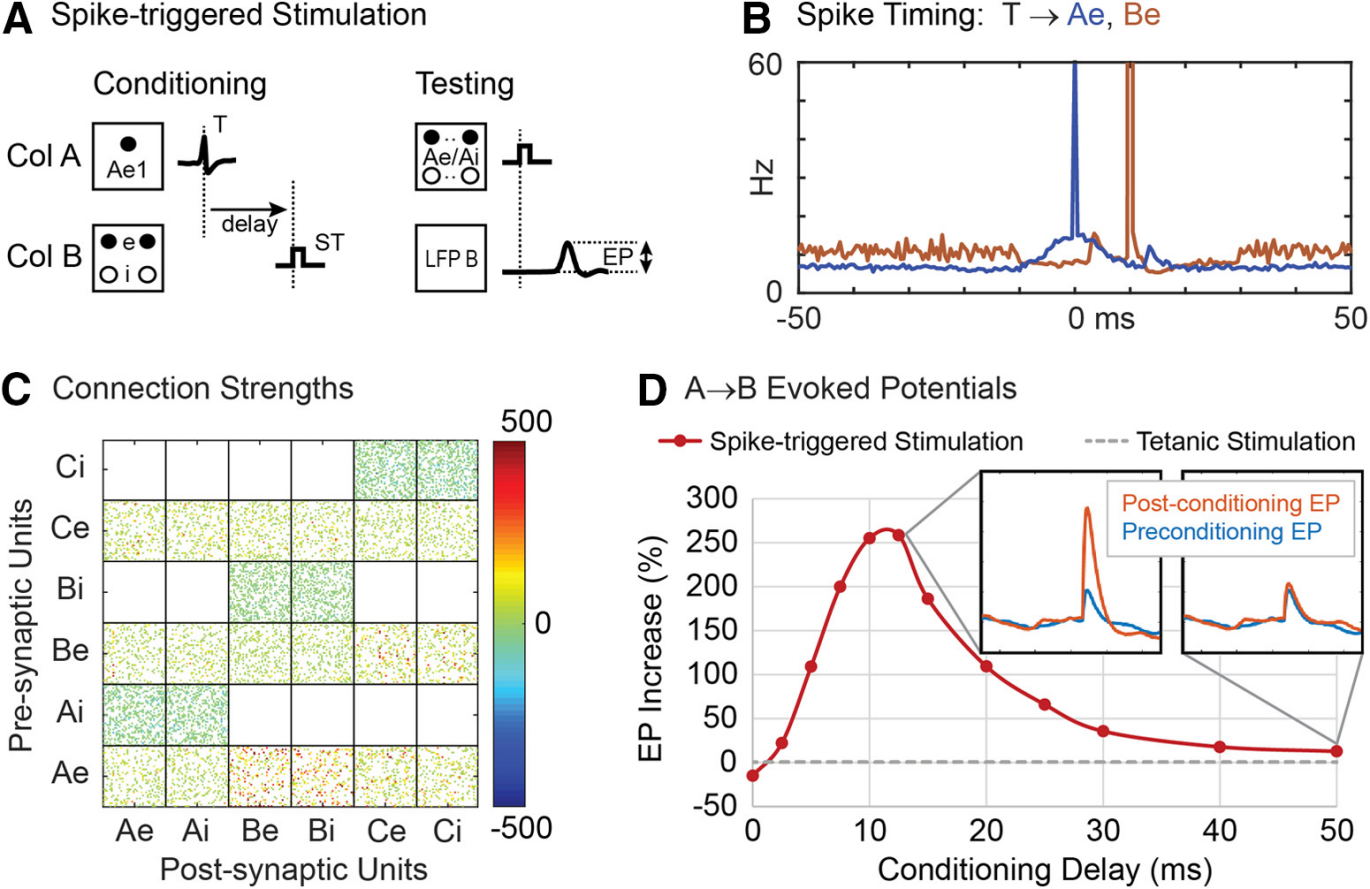
Spike-triggered stimulation. ***A***, A Conditioning stimulus (ST) is applied to all units in column B at a delay from each trigger spike (T) detected on the first excitatory unit (Ae1) of column A. Testing shows stimulus applied to all column A units to evoke a potential (EP) in LFP of column B. ***B***, Histogram of excitatory A and B unit firings aligned with spikes on Ae1 (T) during conditioning with a 10-ms delayed stimulus. ***C***, Final connection strengths (calibrated in μV) between units after conditioning. ***D***, Average percent increase in the EP as a function of the delay between trigger spike and stimulation. Insets show average EPs before and after conditioning for two delays. Gray dashed line shows the EP response in column B after tetanic stimulation of column B.

To simulate conditioning with spike-triggered stimulation (Jackson et al., 2006; Rebesco et al., 2010), each time the first excitatory unit (Ae1) in column A fired, a conditioning stimulus was applied to all units in column B at a given delay (Fig. 3*A*). Conditioning effects were observed as increased connection strengths between column A and column B. Figure 3*C* shows color-coded strengths of all connections after spike-triggered stimulation with a spike-stimulus delay of 10 ms, showing strengthened connections from Ae to B units compared with connections between other columns. Connectivity between columns A and B was also documented by the average EP (Fig. 3*D*, insets) in the LFP of column B produced by a test stimulus to A (Fig. 3*A*).

After conditioning for 500 s of simulation time, the conditioning unit (Ae1) showed near maximum connection strengths to its target units in column B. Other units in A also show increased connections to B depending on the percent of correlated bias inputs to A. Networks with 30% correlated bias inputs show a moderate conditioned response in other column A→B connections. Networks with 20% correlated bias inputs show little conditioned response except for connections from unit Ae1. Figure 3*B* shows the average firing rates of the Ae and Be populations relative to the triggering Ae1 spike times (T). The sharp peak at 0 ms represents the trigger spikes and the broad peri-trigger peak reflects the synchrony between Ae units imposed by 30% external correlated bias. Figure 3*B* also shows the times of spikes in column B (red); the large peak at 10 ms reflects the occurrence of stimulus pulses in B.

Figure 3*D* shows the size of A→B EPs after spike-triggered stimulation at various spike-stimulus delays. The peak in the curve reflects the effect of the STDP rule. At long delays, the spike-triggered conditioning effect approaches the small effect of tetanic stimulation applied to column B (dashed line). At “zero” delay the size of the EP is decreased (Fig. 3*D*). In this case the spike-stimulus delay is shorter than the conduction delay and connections from A to B become weaker. This decrease in synaptic strength is consistent with the STDP rule and with experimental results obtained for corticospinal connections (Nishimura et al., 2013).

**Figure 4.**
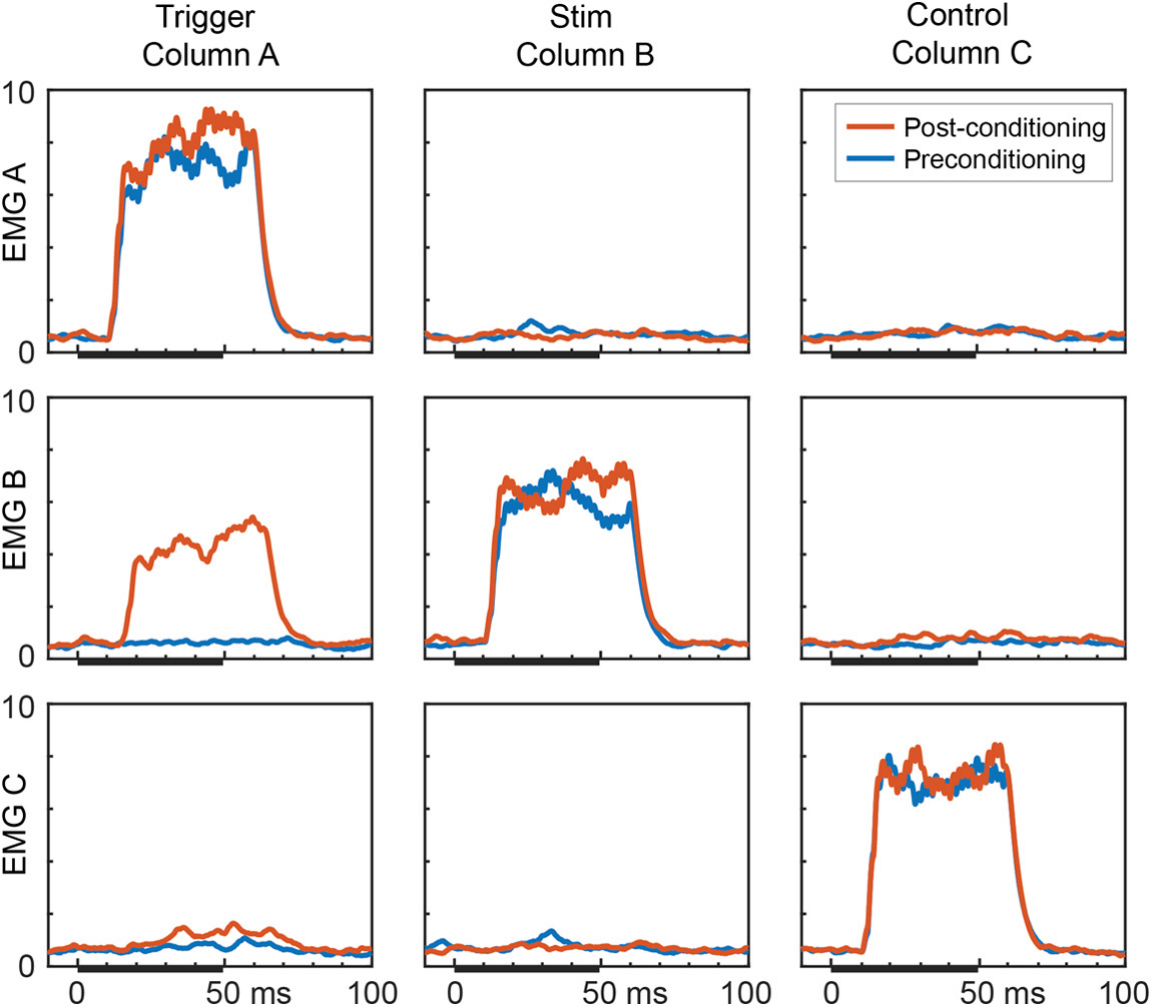
Simulation of output effects on muscles before and after spike-triggered stimulation. Averages of rectified EMG responses evoked by repetitive stimulation of column A (containing conditioning trigger unit, left), column B (stimulus column, middle), and column C (control, right) for preconditioning (blue) and postconditioning (orange) periods (compare Jackson et al., 2006; Fig. 2) For this simulation, intercolumn connection probability was doubled to 1/3, and the correlated bias drive was set to 50%. The test stimulus train was 1 mV, 25 pulses, 500 Hz delivered during intervals marked by black bars (the response delay is due to the 10-ms corticospinal conduction time). Ordinate scale is in millivolts.

The original experiments of Jackson et al. (2006) could not document the conditioning effects by recoding short-latency EPs because of stimulus artifacts, so instead measured conditioning effects indirectly by using cortical microstimulation to evoke EMG responses. To simulate these experimental observations, the motor unit pools were activated by trains of cortical stimuli delivered at separate times to each column (Fig. 4). This simulation shows that after conditioning, stimulation of column A now also evoked responses in the muscle of column B, mediated by the strengthened A→B connections and as reported by Jackson et al. (2006; their Fig. 2).

### EMG-triggered stimulation

**Figure 5.**
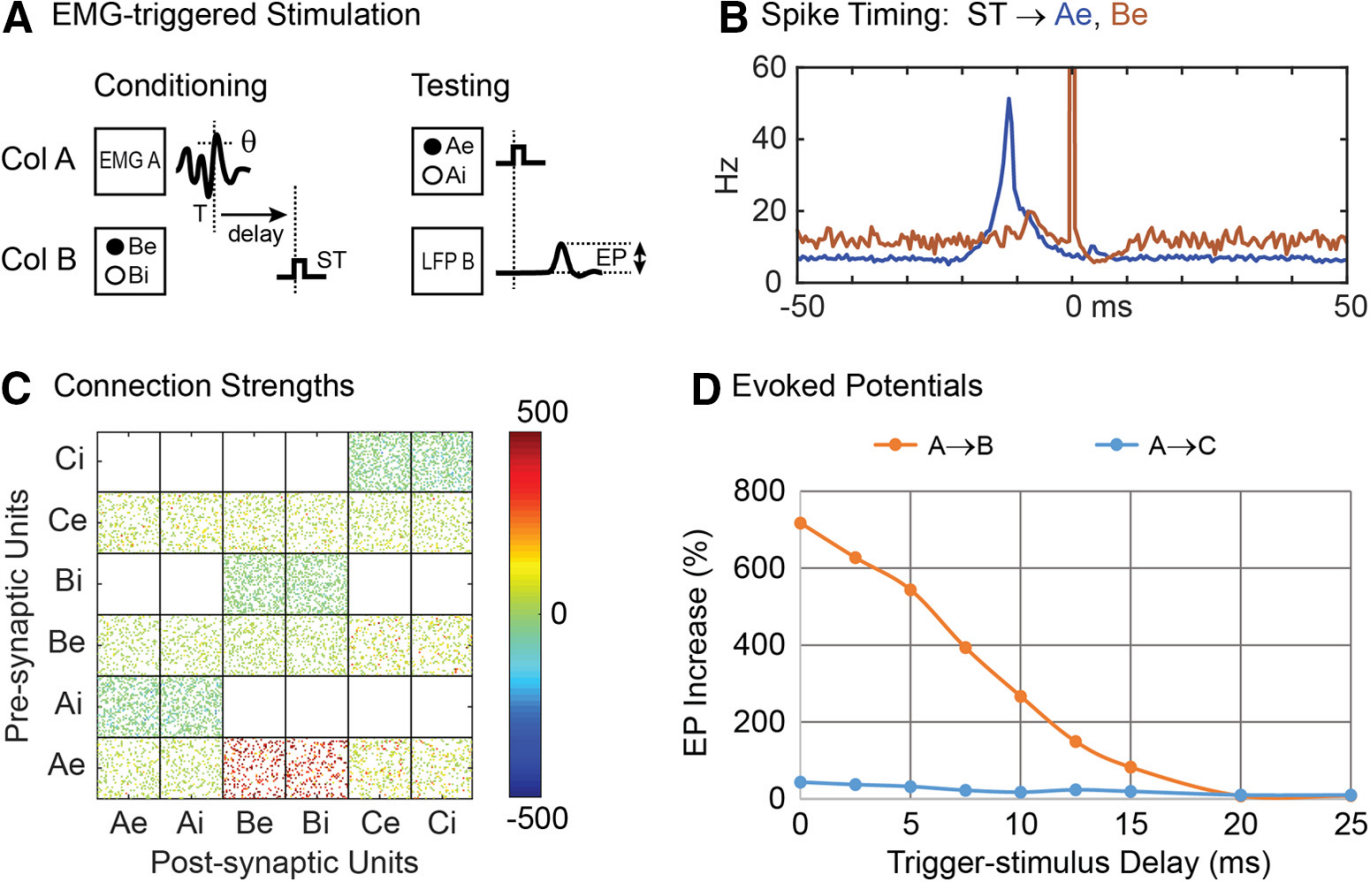
EMG-triggered stimulation. ***A***, A conditioning stimulus is applied to all units in column B at a delay after threshold crossing (θ) in EMG of A motor units. ***B***, Firing rate histograms of Ae and Be units during conditioning aligned with EMG A threshold detection and stimulation (0 ms). ***C***, Connection strengths between presynaptic and postsynaptic units after conditioning. ***D***, The average percent increase in the EPs evoked in column B (orange) and column C (blue) from stimulating A as function of delay between EMG threshold crossing and delivered stimulation.

Cortical conditioning effects could also be produced in NHPs by triggering cortical stimulation from muscle activity (Lucas and Fetz, 2013). To simulate these experimental results the trigger pulses were obtained from threshold crossings of multiunit EMG activity of muscle A (Fig. 5*A*). The threshold was chosen such that triggered stimuli were delivered to column B at a rate comparable to that used for other conditioning methods (∼3000 conditioning stimulations over a 500-s conditioning period). This protocol strengthened the synaptic connections from A to B, as shown by the connectivity matrix (Fig. 5*C*) and by the A→B EPs (Fig. 5*D*). The conditioning effect is explained by the computed histogram of spikes in the Ae units aligned with the EMG threshold detection (Fig. 5*B*). This shows a peak in firing of A units (blue curve) that generated the coincident input to the motor units and that preceded the triggered responses in B (red peak). The delay between these peaks represents the 10-ms cortico-motor-unit conduction time plus a minor effect of EMG rise to trigger threshold.

### Paired-pulse stimulation

**Figure 6.**
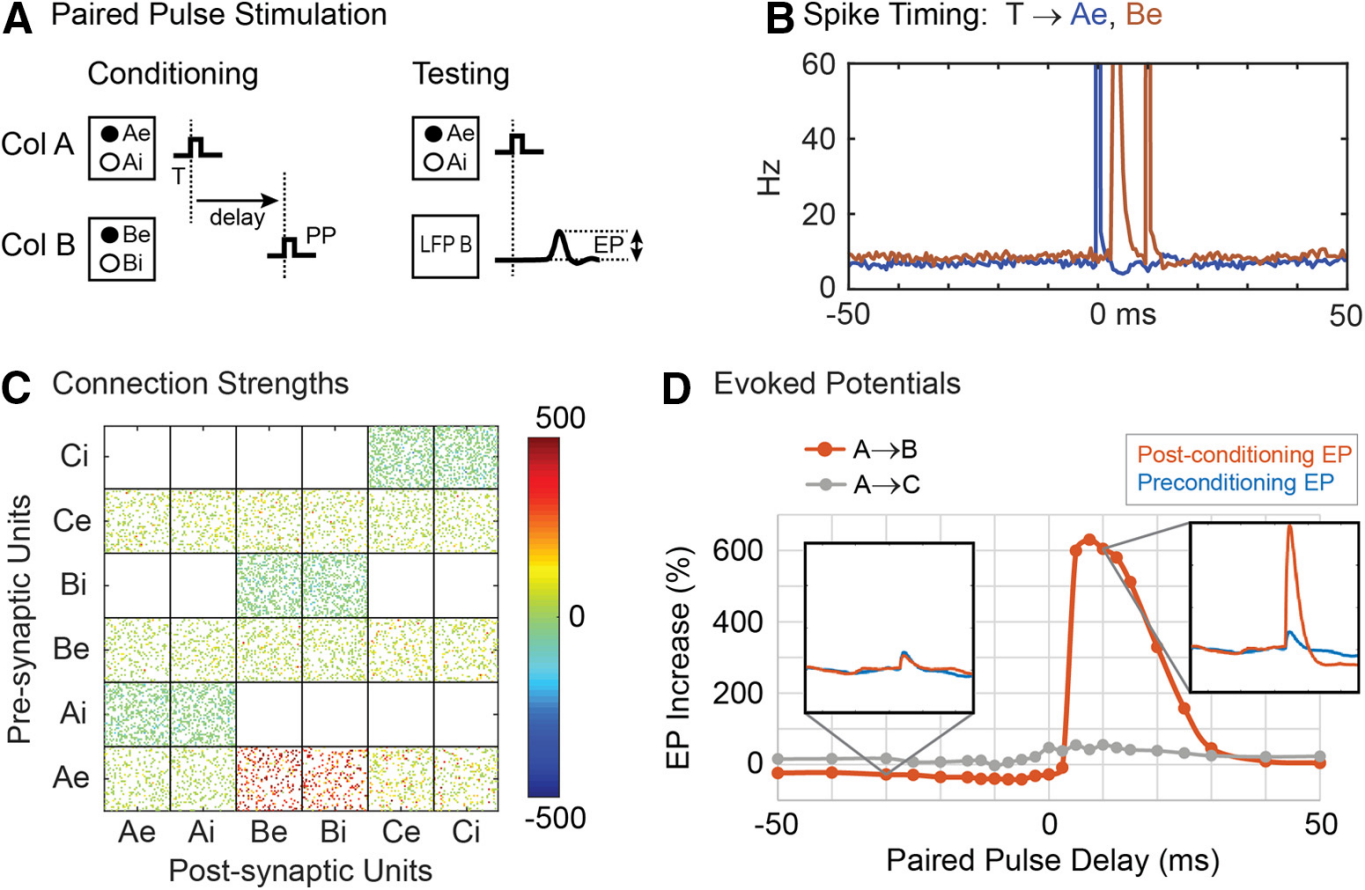
Paired-pulse stimulation. ***A***, Pairs of pulses delivered sequentially to A and B with fixed delay. ***B***, Firing rate histograms of Ae and Be units during conditioning aligned with times when the first of the paired stimuli occurred (10-ms conditioning delay). ***C***, Connection strengths after conditioning. ***D***, Average percent increase in the EP between the conditioned and unconditioned effect as a function of the interstimulus delay. Insets show the shape of the average EP at conditioning delays of −30 and 10 ms. Gray trace plots same for EPs in column C.

Paired pulse conditioning (Rebesco and Miller, 2010; Seeman et al., 2017) was simulated by stimulating column A followed by stimulation of column B at a fixed delay (Fig. 6*A*). The resultant connection strengths are shown in Figure 6*C* for a stimulus delay of 10 ms. Conditioning effects were also documented by delivering test stimuli to column A and measuring the average EPs in columns B and C. Figure 6*D* plots the change in amplitudes of the A→B EPs and A→C EPs as a function of interstimulus delay. Consistent with the bidirectional STDP function, there is a decrease in the size of the EPs for negative conditioning delays (i.e., B stimulated before A), but this is shallower than the peak for positive delays because of the choice for the weakening parameter, which tended to keep synaptic weights small. The histograms of unit firings show the peak in A spikes produced by the stimulus in A (blue trace) and two peaks in B firing (red trace): the first peak is due to a synaptically relayed response to the A burst and the second is because of the delayed stimulus of B.

For the same stimulus amplitude, paired pulse conditioning tends to be stronger than spike-triggered conditioning (see below, Testable outcomes) and does not require correlated bias inputs, since the stimulation pulses themselves evoke strong correlated activity between A and B. The conditioning effect is seen with far fewer stimuli than used for spike-triggered stimulation, for the same stimulus intensity. Our 500-s conditioning time yielded only 700 paired pulse conditioning stimuli, compared with over 3000 stimuli when using the spike-triggered stimulation protocol.

In these simulations, conditioning effects were obtained by delivering pairs of single pulses. However, in the physiological experiments it was necessary to use a triplet of pulses to obtain effects (Rebesco and Miller, 2010; Seeman et al., 2017). In the model, conditioning with paired triplets of stimuli shows a more potent conditioning effect, which became evident for a range of lower intensities where single stimuli were insufficient (Fig. 10).

### Cycle-triggered stimulation

**Figure 7.**
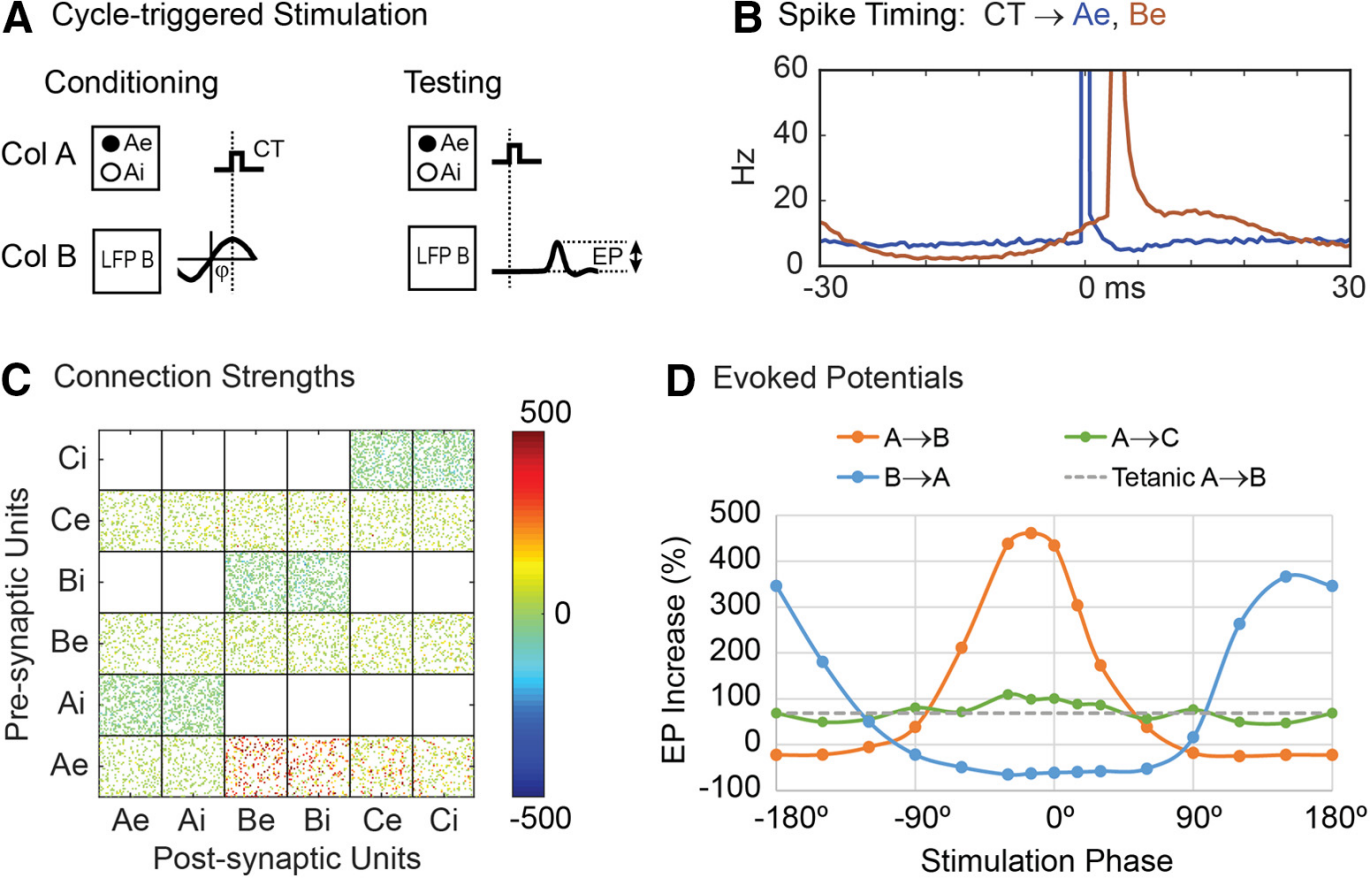
Cycle-triggered stimulation. ***A***, A stimulus is applied to column A at a particular phase of LFP oscillations in column B. ***B***, Peristimulus time histogram of Ae and Be units during cycle-triggered stimulation at 0° phase. ***C***, Connection strengths after conditioning (0° phase). ***D***, Increase of the evoked responses as a function of stimulation phase. Legend identifies traces for stimulated and recorded columns. Dashed line shows A→B EP increase for tetanic stimulation on column A with a rate approximately matching that for cycle-triggered stimulation.

To simulate cycle-triggered conditioning (Zanos et al., 2018), episodes of oscillatory beta activity were generated by modulating the bias input to column B (episodes of six oscillations at 20 Hz occurred four times during each 10-s simulation time block). The LFPs from column B were filtered with a 15- to 25-Hz band pass and when this filtered LFP exceeded a given level, the next zero crossing was either taken as a 0° phase (when rising through zero) or 180° phase (falling through zero) to define the phase of the cycle trigger. During conditioning a stimulus was applied on all units in column A whenever the cycle trigger for the specified phase occurred in B (Fig. 7*A*). To measure conditioning effects, we applied test pulses on column A before and after the oscillatory episodes, as in the original experiments. These test pulses could cause a certain amount of tetanic conditioning, but this was lower than the changes caused by the cycle-triggered stimulation.

As shown in Figure 7*C*, cycle-triggered stimulation at 0° phase shift increased connections from column A→B and to a lesser extent the connections from column A→C. In addition, the B→A connections were reduced. Cycle-triggered stimulation at 180° phase shift decreased connections from column A→B and increased connections from column B→A (Fig. 7*D*).

Figure 7*B* shows histograms of unit firings relative to the trigger for stimuli delivered at 0° phase shift. The Ae units fired in response to the stimuli (peak at zero in blue curve). The Be units (red) show the broad oscillatory increase and response to the stimuli.

Conditioning was also performed with stimuli delivered at other phases. Figure 7*D* shows how evoked responses vary with stimulation phase for A→B, A→C, and B→A connections. Maximum evoked responses for A→B occurred at about −15°, which matches the phase difference between the bias input modulation and the resulting modulation in the filtered LFP of column B. Maximum B→A EP responses occurred at approximately 150° phase shift. The A→C connections showed a very weak modulation, which is consistent with experimental results for control sites whose LFPs were not entrained with B.

### Control for tetanic stimulation

**Figure 8.**
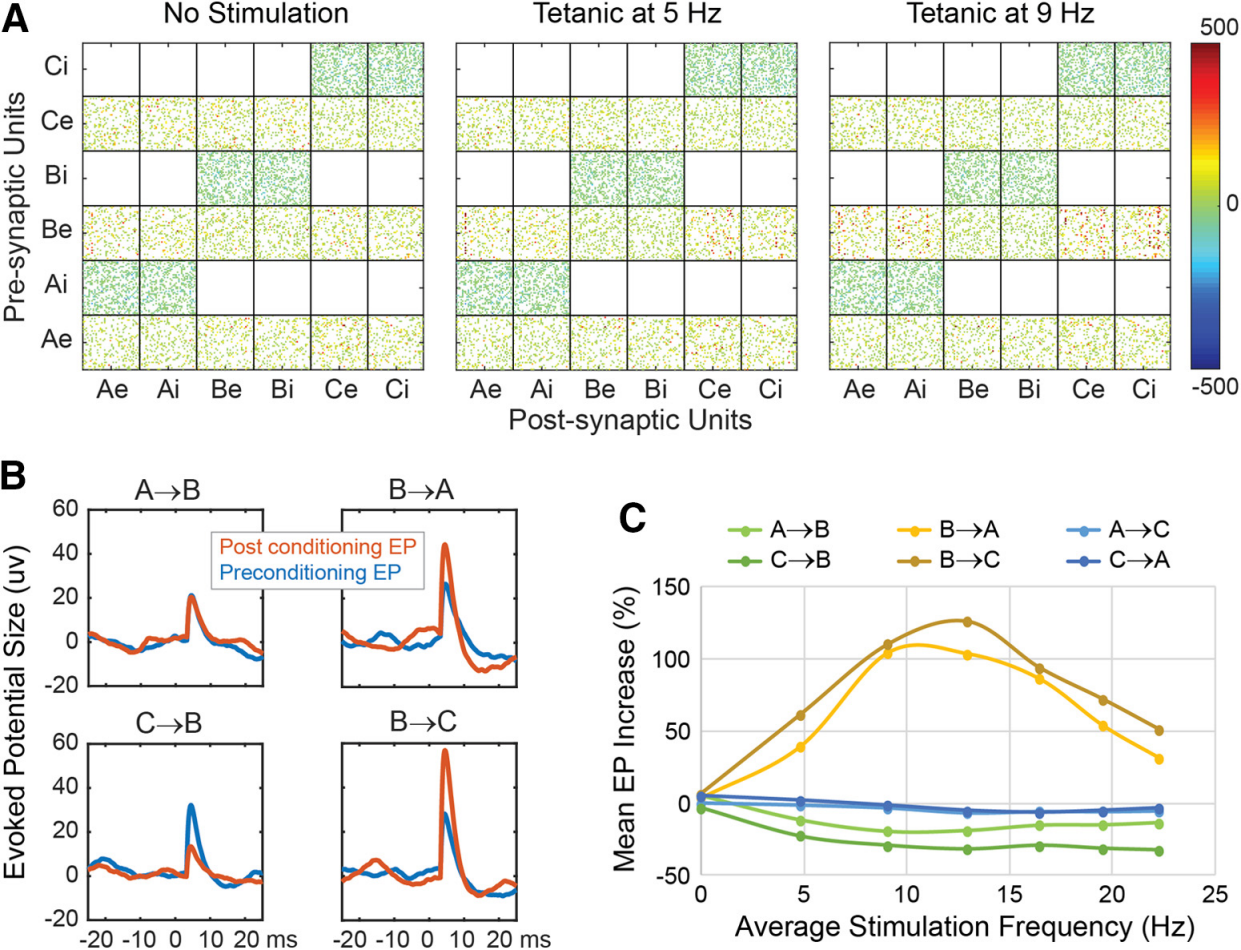
Tetanic stimulation. ***A***, Effect on connection strengths of tetanic stimulation of column B at 5 and 9 Hz (intensity of 2.0 mV), compared with no stimulation. ***B***, Examples of averaged EPs before and after tetanic stimulation of B at 9 Hz. ***C***, Mean (over 10 randomized networks) of averaged EP changes produced by tetanic stimulation of B at different frequencies. Legend identifies traces for test-stimulated → EP-recorded columns.

An important control for conditioning effects is the effect of a comparable amount of open-loop stimulation not triggered by preceding activity. To reduce the effects of such tetanic stimulation, the amount of external bias drive and the area difference between the weakening and strengthening sides of the STDP curve were chosen such that tetanic stimulation, at rates similar to rates for conditioning protocols, did not cause large conditioning effects. The results of tetanic stimulation are shown in Figure 8, for Poisson-distributed stimulation delivered to column B during the conditioning period. The connection matrices for tetanic stimulation show slight increases over the “no stimulation” network in the connectivity from column B to A and B to C (Fig. 8*A*). The changes in connectivity between columns as measured by EP amplitudes are illustrated in Figure 8*B* and plotted in Figure 8*C* as a function of stimulus frequency. Tetanic stimulation of B has the greatest effect on connections from B for frequencies between 8 and 16 Hz. The EP increases for other connections are small or slightly negative (Fig. 8*B*,*C*). Most of the conditioning protocols modified the A→B connections with triggered stimulation of column B. In these cases, the conditioned A→B effects were clearly larger than the tetanic effect, which had the opposite effect of decreasing A→B EPs. For cycle-triggered stimulation of column A, the conditioned effect was larger than the effect of tetanic stimulation of A alone (Fig. 7*D*).

### Testable outcomes

**Figure 9.**
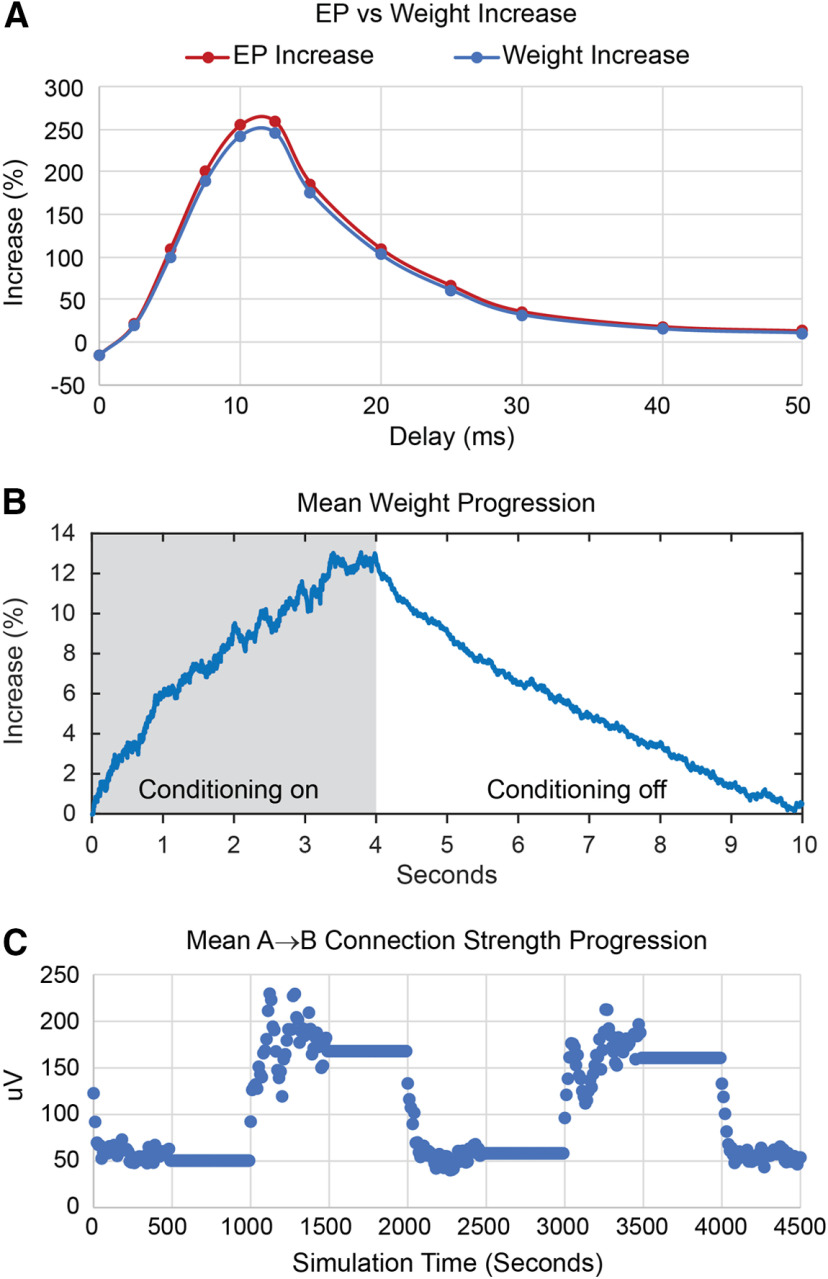
Network weight measurements. ***A***, Comparison between EP increase and mean A→B weight increase for conditioning with spike-triggered stimulation as function of spike-stimulus delay. ***B***, Average evolution of A→B weights during and after 4 s of spike-triggered stimulation at 10-ms conditioning delay (averaged over 50 time blocks). ***C***, Mean A→B connection strengths calculated at the end of each 10-s time block and plotted over two passes through the preconditioning, testing, and spike-triggered stimulation periods.

Importantly, our IF model can simulate variables and procedures that were not documented in the original physiological experiments. Notably, the strength of synaptic connections in the model was documented by computing stimulus-EPs (Figs. 4*D*, 5*D*, 6*D*, 7*D*, 8*C*), a test that could in principle be performed experimentally, but is technically difficult. Another comprehensive measure of conditioning effects on synaptic strength is the sum of all the synaptic weights connecting one column to another. There is no feasible physiological or anatomic experiment to measure these weights directly *in vivo*, but they can be readily calculated from the model. Significantly, Figure 9*A* shows that the computed weight changes closely track the changes in the EPs. Moreover, the weights can be tracked with high temporal resolution, during and after the conditioning to document the induction and decay of the conditioned weight changes. Figure 9*B* shows the time course of the weight changes in the A→B connections during and after a period of spike-triggered stimulation with STDP. The stimulation is applied during the first 4 s of each 10-s time block. The plot of the average time course over 50 blocks shows how the weights tend to increase during the stimulation and then decay toward their preconditioned values after stimulation is turned off. Figure 9*C* tracks these weight changes during and after two spike-triggered stimulation periods to show that weight changes can be induced and decay in a minute or two of simulated time. This figure also shows the variability in conditioning because of the training factor and firing rates of the units. A smaller training factor would reduce the variability of the conditioned weights but would also extend both the training and decay times.

**Figure 10.**
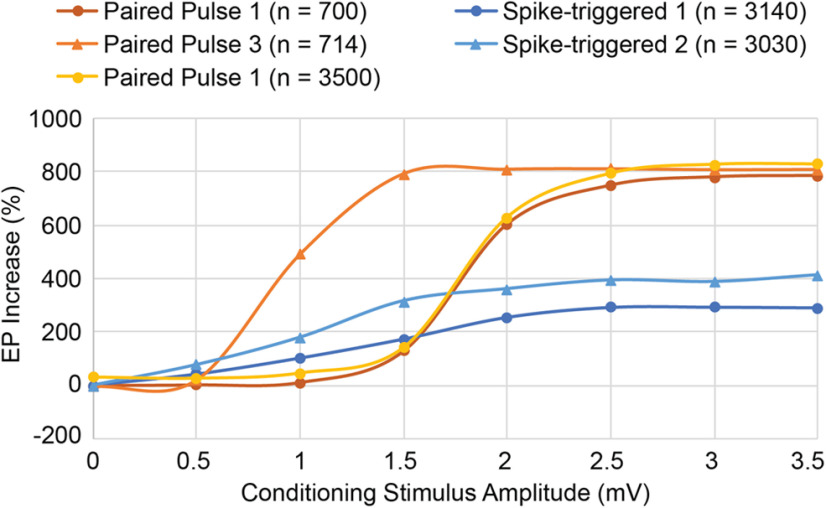
Effect of conditioning stimulus amplitudes and trains. Stimulating with pulse trains (33-ms interstimulus interval) instead of single pulses increases the efficacy of conditioning for spike-triggered stimulation (spike-triggered 2 vs 1) and for paired-pulse stimulation (paired pulse 3 vs 1) at all stimulus amplitudes. *n* is the total number of stimuli delivered during each simulation. Running the paired pulse 1 conditioning longer (*n* = 3500 compared with *n* = 700) does not provide the same increase in efficacy as paired pulse 3 (*n* = 714) until efficacy approaches an upper limit at stimulus amplitudes ≥2.5 mV.

Another testable property that the model can predict is the relative efficacy of conditioning as a function of stimulus intensity and number, as shown for the spike-triggered and paired pulse protocols in Figure 10. The curve for spike-triggered stimulus pairs rises faster than the curve for spike-triggered single stimuli; this is partly because of the pairs acting as a higher intensity stimulus, but that does not entirely explain the higher asymptote achieved. The curve for paired single-pulse stimulation initially rises more slowly than that for spike-triggered single stimuli, but then surpasses it at 2 mV, our standard conditioning simulation intensity. The curve for paired triplet stimuli (which had to be used experimentally) rises much faster than that for paired single-pulse stimulation, revealing a stimulus range over which the triplets are more effective than single pulses. The curves asymptote at high stimulus intensities when most of the units in the stimulated column respond.

### Testable predictions for novel protocols

**Figure 11.**
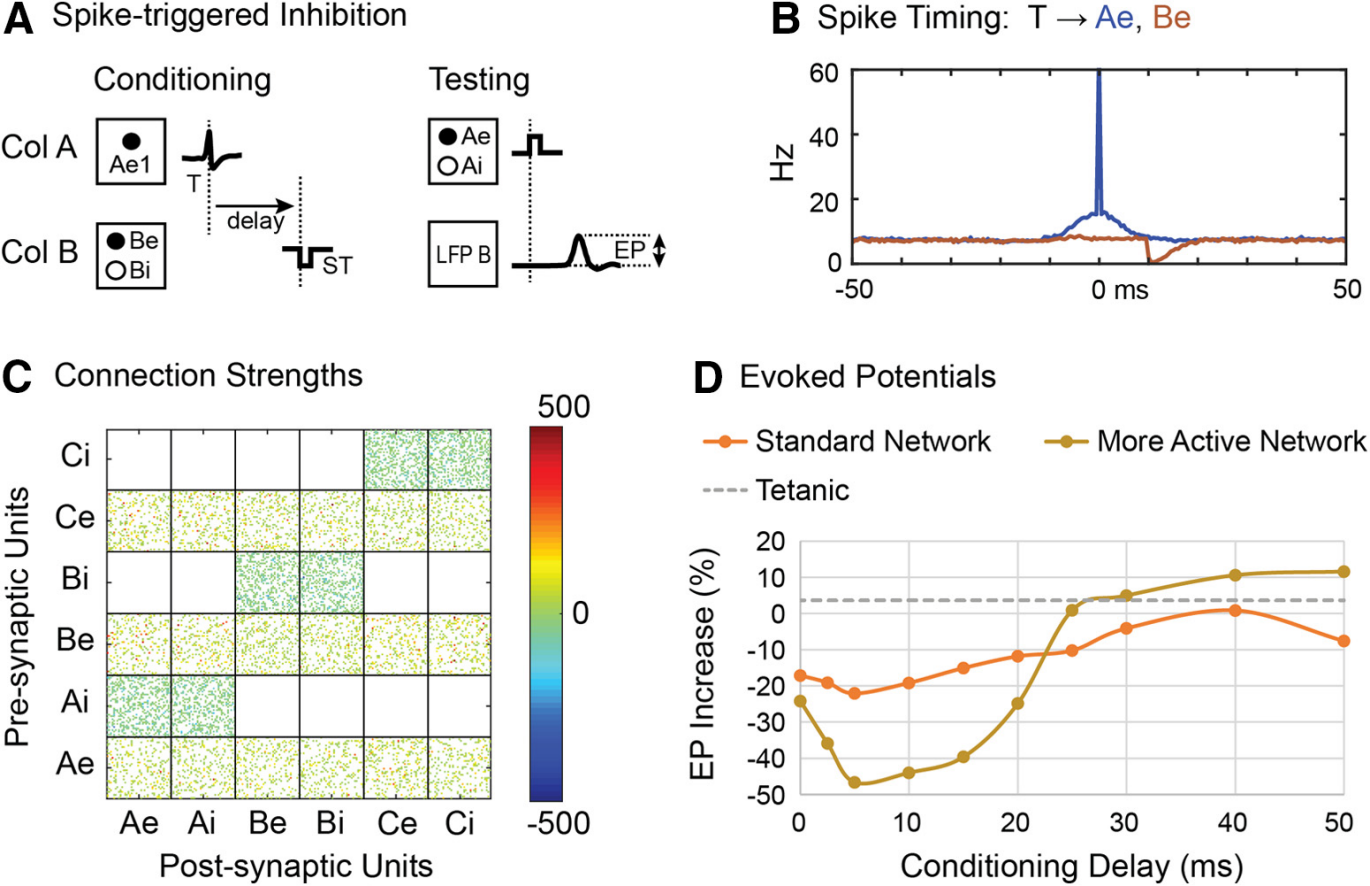
Spike-triggered inhibition. ***A***, Spikes in one unit in column A (Ae1) trigger an inhibitory effect on all units in column B. ***B***, Perispike histogram of Ae and Be units. ***C***, Postconditioning connectivity matrix for 10-ms delay. ***D***, The standard network parameters yield a relatively mild EP decrease (orange curve). A more active network with 45% correlated bias drive, a weakening value of 0.5, and a −3-mV stimulus produces a more robust poststimulus inhibition (brown).

Our IF model can also be used to predict outcomes of experiments not yet performed. For example, it is possible to trigger a pulse of inhibition of column B from action potentials of a cell in column A, which could be achieved by optogenetic spike-triggered inhibition (Fig. 11*A*). The inhibitory effect was modeled as a negative deflection (−2 mV) to the slow decaying part of the membrane potential. The results of this simulation show that the connections from A to B would be reduced (Fig. 11*D*). The trigger-aligned histograms in Figure 11*B* document the pairing of increases in Ae units with subsequent decreases in B units. The time course of conditioned reduction in connections as a function of spike-pulse delay roughly resembles the inverse of the increase with spike-triggered stimulation.

**Figure 12.**
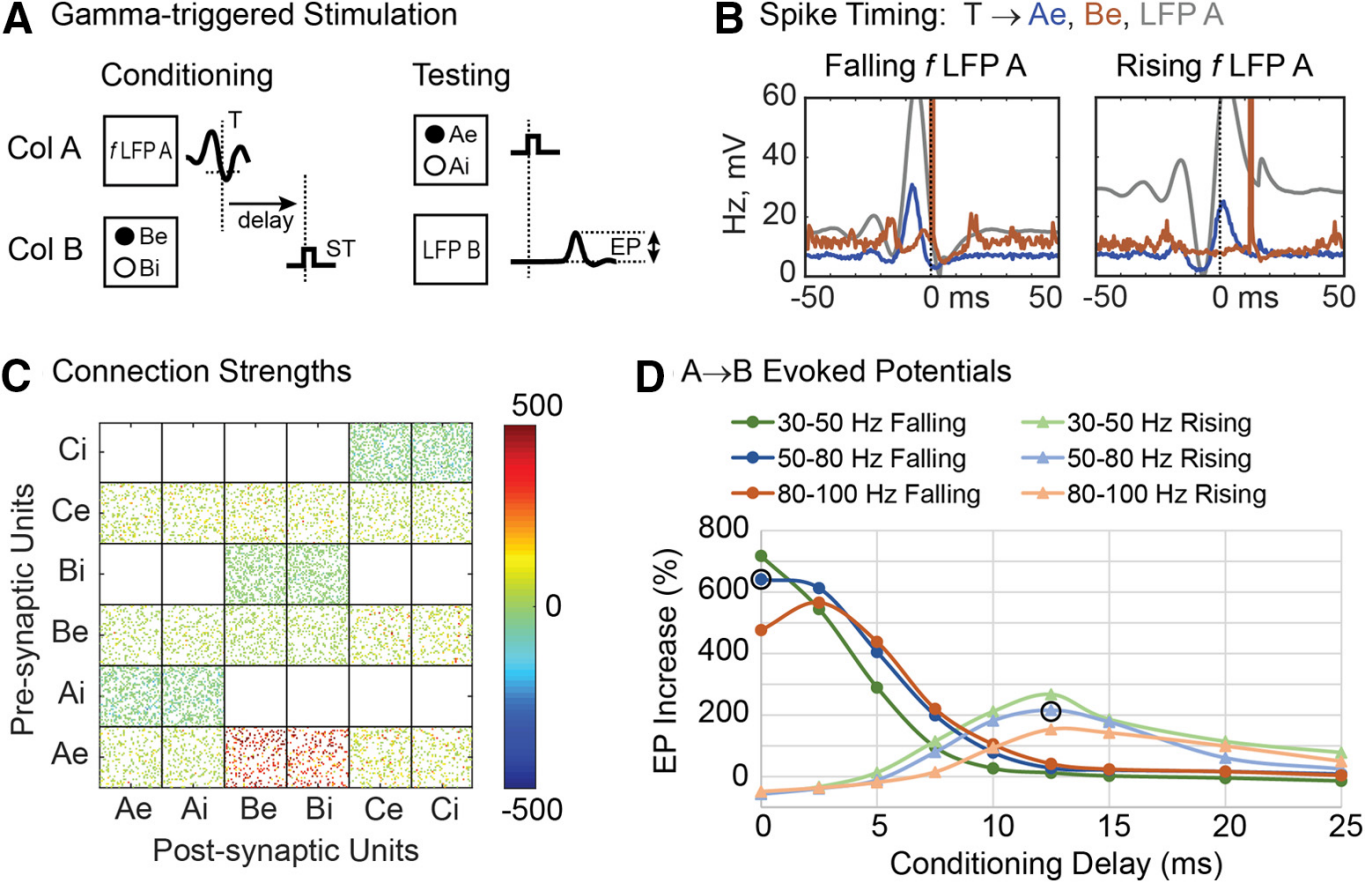
γ-Triggered stimulation. ***A***, Stimuli were triggered from threshold crossings of LFP filtered in the γ frequency ranges shown. ***B***, Firing rate histograms of Ae units (blue) and Be units (red) relative to triggers from falling and rising mid-range γ filtered LFP in A. Gray curve shows simultaneous LFP in A. ***C***, Connection strength matrix for falling LFP filtered in mid γ range (50–80 Hz). ***D***, changes in EPs for rising and falling threshold crossings for different γ frequency ranges. Circled points used for histograms in ***B***.

A second untested conditioning protocol involves triggering stimuli from threshold crossings of *γ* range LFPs. On the rationale that γ LFP reflects multiunit activity, these threshold crossings should detect coincident spiking activity that should also produce plasticity. Figure 12 shows the effects of stimuli in B triggered from band-passed mid-γ LFP in column A (in 50- to 80-Hz range, using 30% correlation in the bias spikes). The histogram of Ae unit spikes aligned with the falling γ threshold crossings (Fig. 12*B*) shows a robust prestimulus peak in Ae units (and in LFP A) that generates the threshold crossing. The increase in A→B connections is shown in both the connection matrix (Fig. 12*C*) and in the size of EPs (Fig. 12*D*). Similar effects were found for triggers from high (80–100 Hz) and low range (30–50 Hz) γ (Fig. 12*D*). Triggering from the rising slope of the γ activity is associated with a later peak in Ae activity (Fig. 12*B*), consistent with a later latency and a smaller conditioning effect (Fig. 12*D*) than triggering from the falling slope.

### Relative efficacy of conditioning protocols

**Figure 13.**
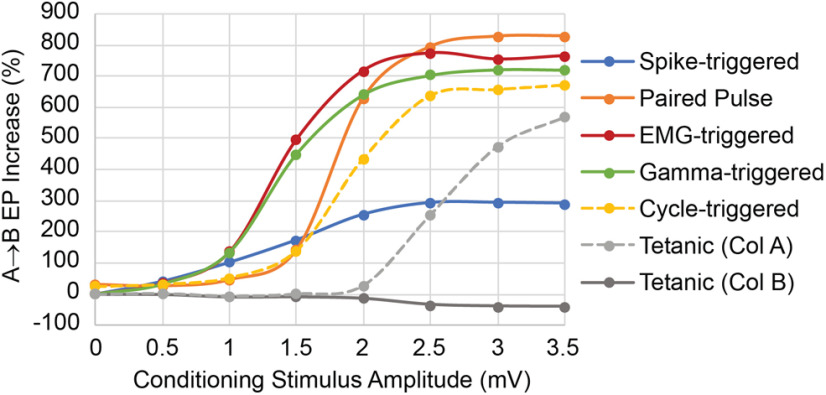
Relative efficacy of conditioning protocols. Curves show increase in A→B EP as function of stimulus intensity using standard network parameters. Tetanic stimulation was randomly spaced with rates approximating that for spike-triggered stimulation. Paired pulse interval was 10 ms, same as the spike-trigger delay, while rising EMG and falling γ-trigger delay was 0 ms.

To compare the relative efficacy of different conditioning protocols, the resulting change of the A→B connections was tested by the change in the EP in B evoked by stimulating A. The results, plotted for different amplitudes of the conditioning stimuli, are shown in Figure 13 (the standard amplitude was 2 mV). These simulations were run with the standard 30% correlated bias drive to each column. As a control for stimulation alone, the solid gray curve shows the effects of tetanic stimulation of column B with exponentially distributed stimulation intervals, at the same average rate as the spike-triggered stimulation (blue curve). Above 1.5 mV, the other four protocols all produce stronger effects than spike-triggered stimulation, probably because the stimuli involve larger numbers of Ae spikes. In particular, triggering from EMG and γ produce larger effects than spike-triggered stimuli above 1 mV because they are selective for coincident Ae spikes (as shown in the histograms in Figs. 5*B*, 12*B*).

Tetanic stimulation of column B (gray solid curve) is the control for spike-triggered, γ-triggered and EMG-triggered stimulation of B. Tetanic stimulation of column A (gray dashed curve) is the control for cycle-triggered stimulation of A (here performed at a phase of 0° in β cycles). Both tetanic curves are relevant to paired-pulse stimulation.

The conditioning effects asymptote at stimulus intensities above 2.5 mV because of maximal activation of units in the stimulated column. The asymptote for spike-triggered stimulation (blue curve) is lower than those of the other conditioning protocols, but this was a function of the synchrony input driving the Ae units and could be increased by increasing the amount of synchronous relative to asynchronous input drive.

### Strengthening disynaptic pathways

**Figure 14.**
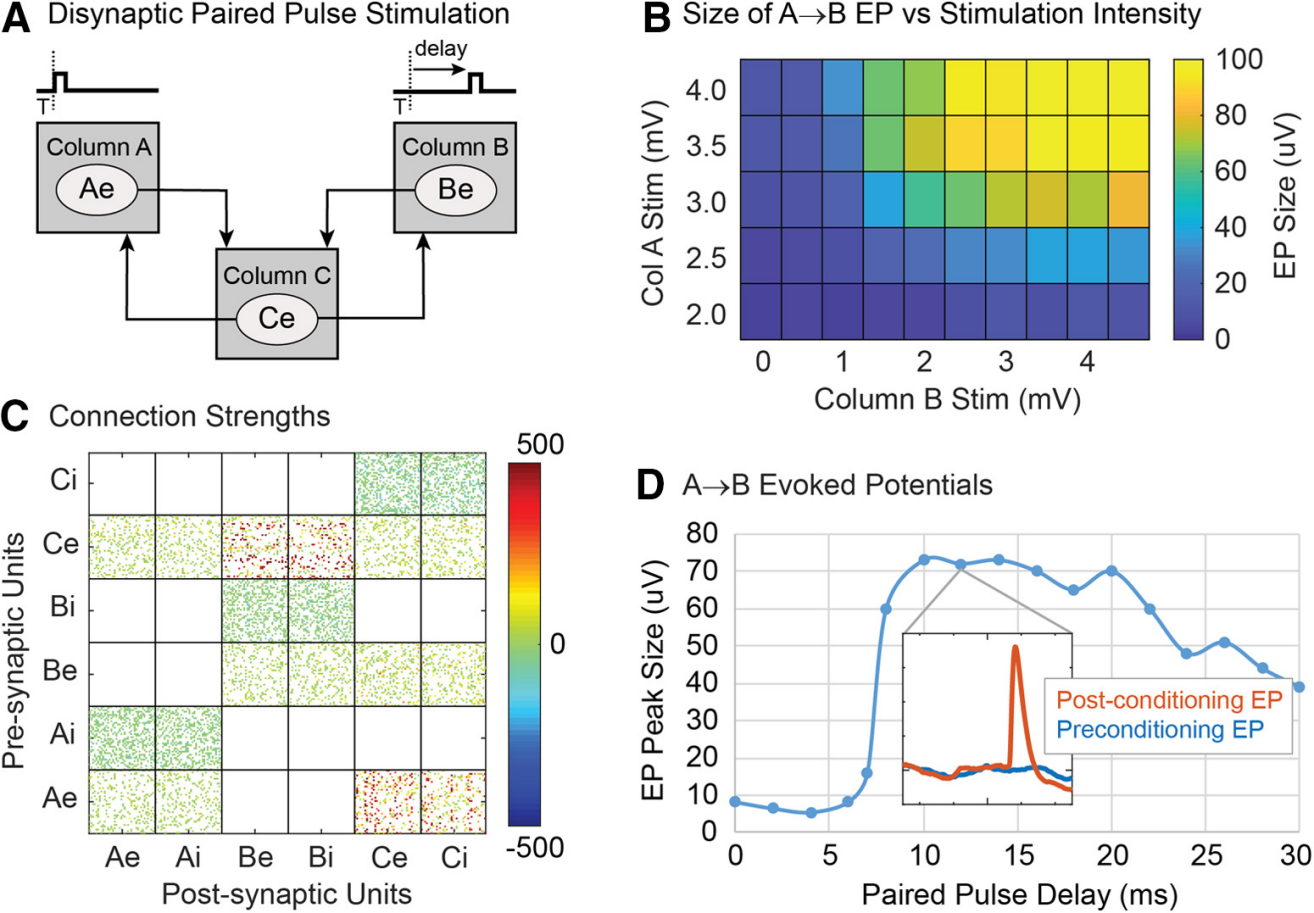
Disynaptic paired-pulse stimulation. ***A***, Circuit connections between columns with standard A↔B connections removed. ***B***, Size of A→B EP as a function of stimulus intensities for the two pulses (for delay of 12 ms). ***C***, Postconditioning connection matrix (for A and B stimulus intensities of 3 mV and delay of 12 ms). ***D***, Size of conditioned A→B EPs as a function of interstimulus delays (A and B stimulus intensities of 3 mV).

The preceding conditioning protocols focused on changes induced in monosynaptic connections. The degree to which polysynaptic pathways can be modified would be of basic interest and clinical relevance for inducing targeted plasticity to treat lesions. Disynaptic conditioning was investigated for paired stimulation of A and B in the absence of direct connections between A and B (Fig. 14). The conditioning protocol is identical to that shown in Figure 6*A* for paired stimulation of intact networks, but now the most direct pathway between A and B is disynaptic, via column C (Fig. 14*A*). The weight matrix shows clear increases in A→C and C→B connections after conditioning (Fig. 14*C*). This increase in the disynaptic path was a function of the intensity of the two stimuli, as shown by the increases in A→B EP size plotted for different combinations of intensity in Figure 14*B*. To produce conditioned changes with pairs of single pulses the stimulation intensity of A had to be at least 2.5 mV, higher than the typical 2 mV used in other simulations. This was likely necessary so that some tetanic conditioning from A→C would occur (as in Fig. 13, tetanic column A). However, tetanic stimulation of A alone did not produce a sizeable disynaptic EP (Fig. 14*B*, B stim = 0). The A→B EPs increase appreciably for conditioning with interstimulus delays above 8 ms (Fig. 14*D*), about twice the minimal delay of 4 ms for direct connections (Fig. 6*D*).

Although larger than standard stimuli were necessary to produce disynaptic conditioning with paired single pulses (Fig. 14*B*), paired triplet stimulation with standard intensities of 2 mV did produce clear disynaptic effects (see below, Fig. 16).

**Figure 15.**
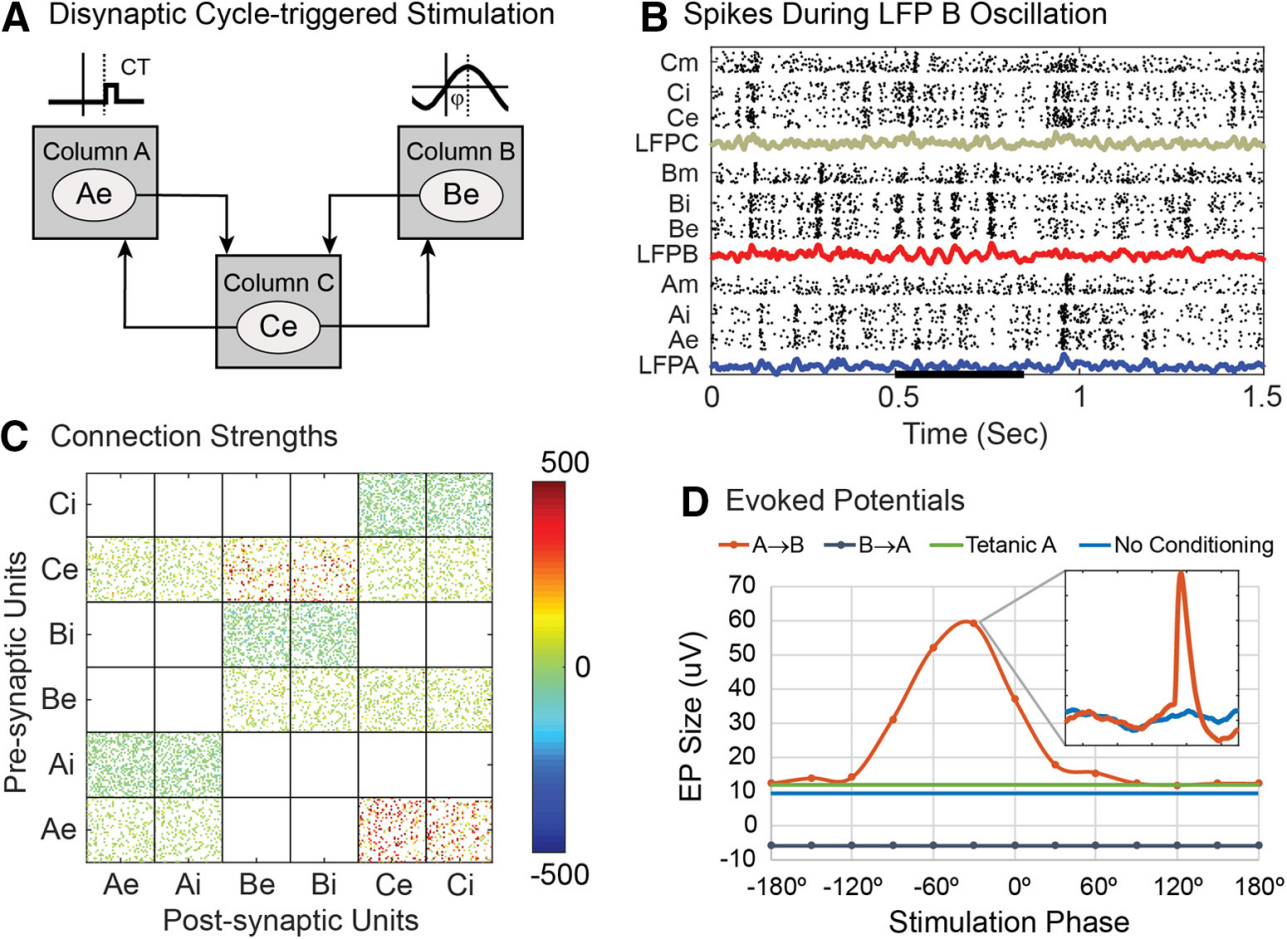
Disynaptic cycle-triggered stimulation. ***A***, Circuit connections between columns A and B are removed. A stimulus (CT) on column A is triggered on phases of an oscillatory episode in LFP B. ***B***, The oscillatory episode in column B (during bar starting at 0.5 s) is produced by modulating the probability of external bias spikes to B with a sine wave (seven cycles at 20 Hz). ***C***, Postconditioning connection matrix shows absence of A↔B connections, and the strengthened A→C and C→B connections that mediate the A→B EP. ***D***, Conditioned EP sizes for conditioning with different stimulation phases (φ). In contrast to cycle-triggered stimulation with full networks, the B→A EP was slightly depressed and unmodulated with phase. Inset shows A→B EP before (blue) and after (orange) conditioning.

Cycle-triggered stimulation could also be used to strengthen disynaptic connections (Fig. 15). The conditioning protocol is identical to normal cycle-triggered stimulation shown in Figure 7*A*, but now connections between column A and column B have been removed (Fig. 15*A*). Therefore, any EP in column B caused by a stimulus in column A must be mediated disynaptically, through column C. Oscillatory drive was applied only to column B (Fig. 15*B*). The weight matrix again shows that conditioning produced clear increases in A→C and C→B connections (Fig. 15*C*). The stimulation intensity had to be increased to 3.5 mV, more than the typical 2.0 mV used in other simulations. This was probably necessary so that some tetanic conditioning from A→C would occur (as in Fig. 13, tetanic column A). However, tetanic stimulation of A alone did not produce a sizeable disynaptic A→B EP (Fig. 15*D*, green line).

Disynaptic A→B connections could not be strengthened with spike-triggered or γ-triggered A→B stimulation, nor with stimulation of B triggered from EMG of muscle A, even with larger than standard stimulus intensities. These protocols did strengthen A→C connections, but they did not produce the necessary increase in C→B connections.

**Figure 16.**
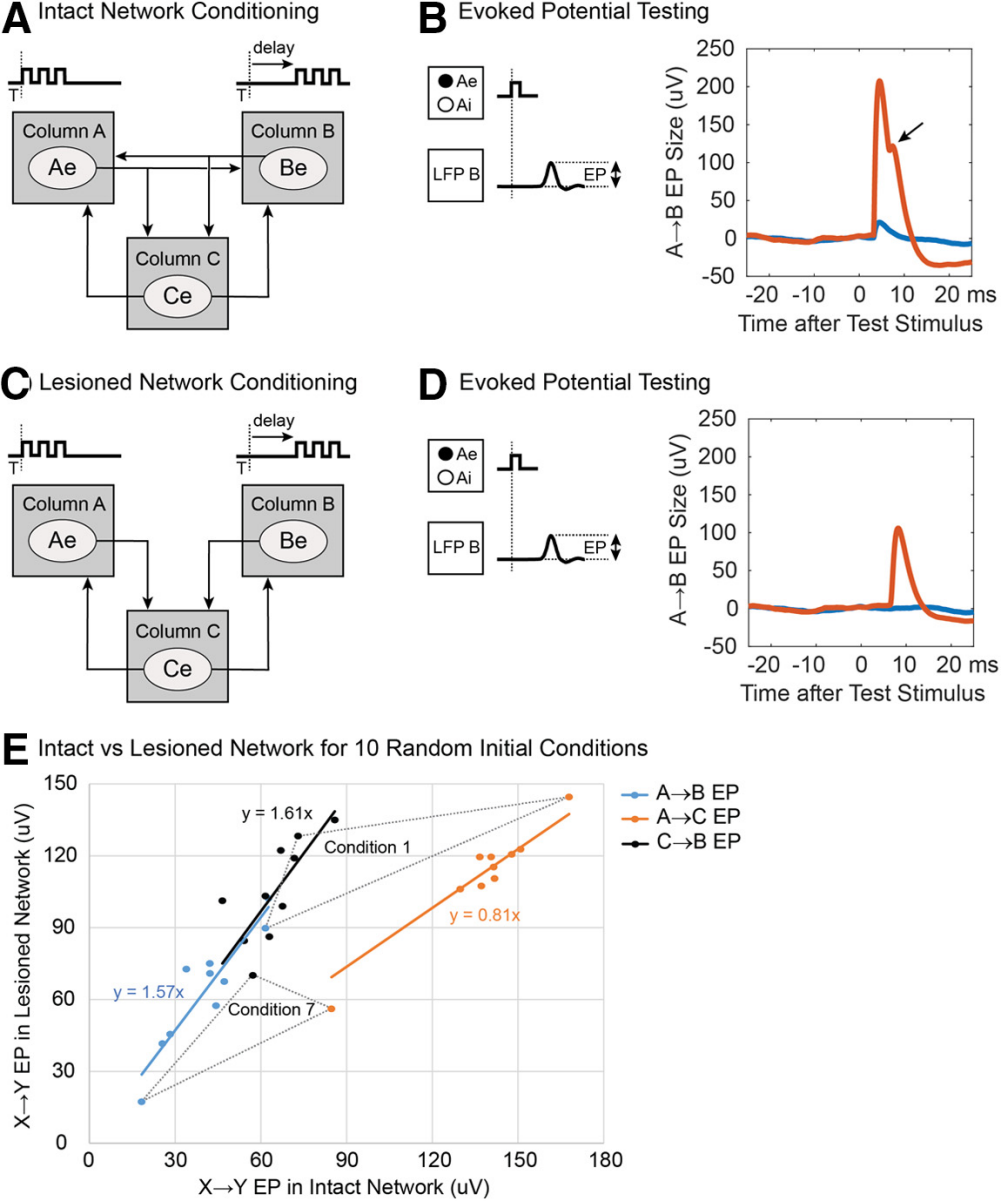
Disynaptic effects with paired triplet stimulation with standard 2-mV stimuli. ***A***, Intact network. ***B***, A→B EP after conditioning the intact network with 10-ms delay between paired triplets (red) and before conditioning (blue). Arrow points to disynaptic component of EP. ***C***, Lesioned network trained without A↔B connections but other connections identical to intact network in ***A***. ***D***, A→B EP after conditioning the lesioned network. ***E***, Sizes of intercolumn EPs in 10 conditioned intact and lesioned networks, with different starting weights. X→Y EP refer to monosynaptic A→C and C→B connections (orange and black points) and disynaptic A→C→B EPs (blue points); for the conditioned intact network, the disynaptic A→C→B EPs were obtained after removing A↔B connections. Dotted triangles connect corresponding EPs from two starting conditions.

The above tests involved disynaptic conditioning with A↔B connections deleted. A related question is whether comparable disynaptic conditioning occurs in intact networks as well. Intact networks conditioned with paired triplets using standard 2 mv stimuli (Fig. 16*A*) showed a clear disynaptic bump in the A→B EP (Fig. 16*B*, arrow). Lesioned networks without A↔B connections that were conditioned with the same paired triplets (Fig. 16*C*) showed a disynaptic A→C→B EP (Fig. 16*D*) whose timing coincided with the delayed bump produced in the intact network. However, their amplitudes differed. To measure the size of the disynaptic EP produced in the intact network we computed the A→C→B EP evoked with the A↔B connections removed for testing. The disynaptic EP produced by conditioning the lesioned networks was ∼1.6 times larger than the recovered disynaptic component of the EP in the intact networks, with the identical conditioning protocol.

To investigate the reason for this difference we examined the sizes of disynaptic A→C→B EPs and the monosynaptic A→B and C→B EPs for the two networks. To elucidate the effects of initial weight choices on these values, this comparison was done for 10 intact networks, each starting with different initial synaptic weights and 10 lesioned networks that had identical initial weights except with A↔B connections deleted before conditioning (with paired triplets). Figure 16*E* plots the relative sizes of these EPs produced in the 10 networks. The disynaptic A→C→B EPs in the intact and lesioned networks (blue points) are proportional, with a linear slope of 1.57. The monosynaptic A→C and C→B connections (orange and black points) were also proportional, with slopes of 0.81 and 1.61, respectively. The two dotted triangles connect three corresponding EPs for two initial conditions (the ones that produced the largest and smallest EPs). The plots illustrate the variation in absolute sizes that can emerge from different starting points and also show that the sizes of the three EPs covary. These plots show that the larger disynaptic EP in the lesioned network can be attributed to a larger increase in the C→B connections.

### Intracolumn connections

Applying the STDP rule to our standard networks tends to suppress connections that are not specifically targeted by conditioning, including recurrent intracolumn connections. This leads to networks in which these connections seem to serve little purpose because similar results can be obtained by completely leaving out inhibitory units and intracolumn connections. To explore how these connections might play a role in conditioning, we ran simulations in which within-column units were strongly interconnected to each other with fixed connections. We found that these networks could show conditioning effects with reduced or no correlated bias input. For example, by fully connecting intracolumn units with connection strengths of 200–300 μV and preventing the STDP rule from modifying them, the various triggered stimulation protocols could mediate conditioning without any external correlated input bias. Moreover, in these networks the inhibitory units do play a functional role in preventing run-away recurrent activity.

**Figure 17.**
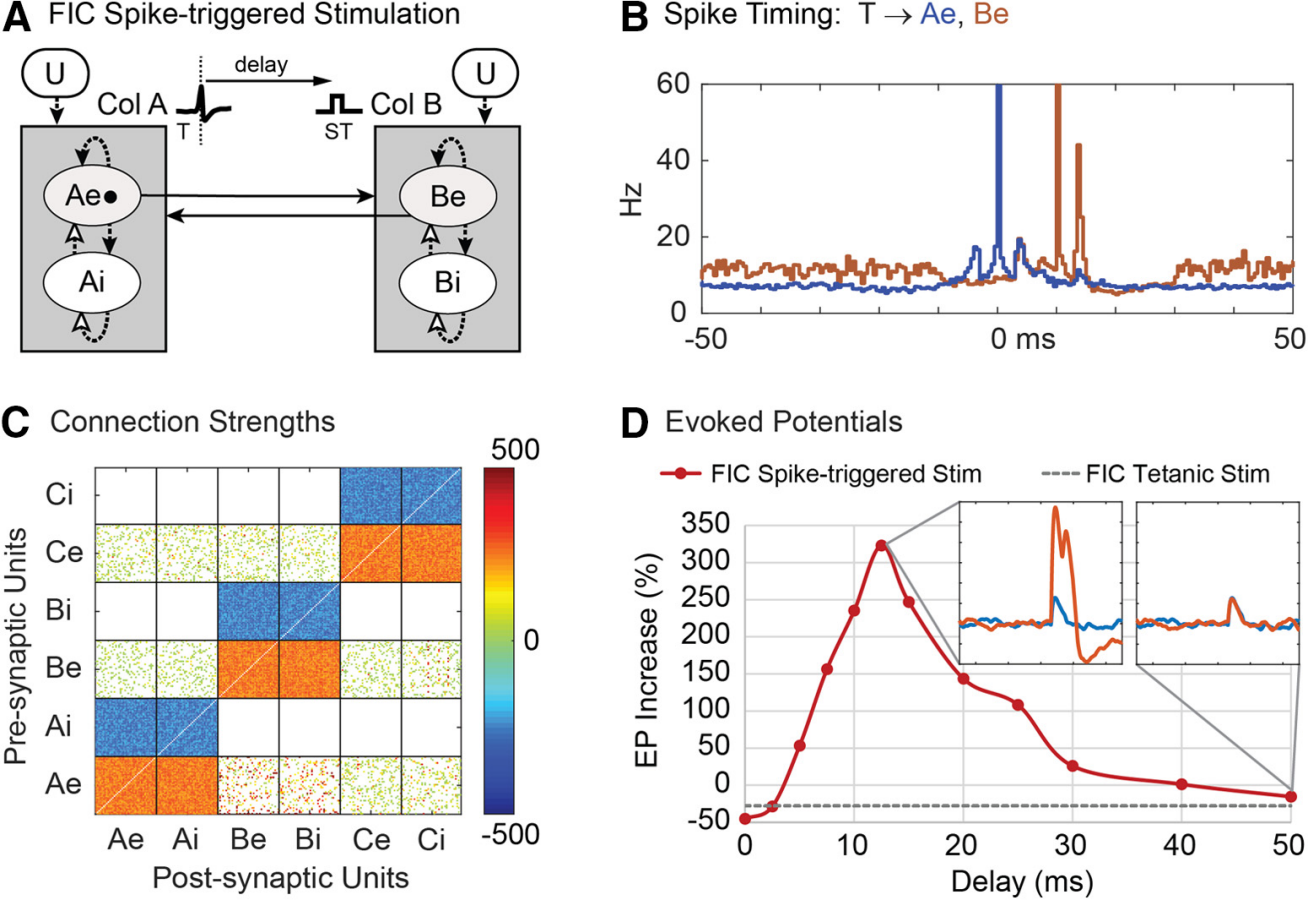
FICs with spike-triggered stimulation. ***A***, A conditioning stimulus (ST) is applied to all units in column B at a delay from each trigger spike (T) detected on the first excitatory unit (Ae1) of column A. Uncorrelated external bias spikes (U) and all intracolumn connections have fixed connection strengths (dashed arrows) leaving only the connections between columns trainable (solid arrows). There are no correlated bias inputs as in previous networks. Column C had similar connectivity (data not shown). ***B***, Histograms of excitatory A and B unit firings aligned with spikes of Ae1 (T) during conditioning with a 10-ms delayed stimulus. Secondary peaks occur because of the FICs. ***C***, Final connection strengths (calibrated in μV) between units after conditioning. ***D***, Average percent increase in the A→B EP as a function of the delay between trigger spike and stimulation. Insets show average EPs before and after conditioning for two delays. Gray dashed line shows the EP response in column B after tetanic stimulation of column B.

Figure 17 shows such a network with fixed intracolumn connections (FICs) operating with the same spike-triggered stimulation protocol used in our standard networks (Fig. 3). However, this FIC network does not employ any correlated bias inputs and instead relies on recurrent activity in column A to create sufficient correlated unit firings to facilitate the conditioning effects. The FIC in column A (Fig. 17*A*, dashed arrows) cause secondary peaks in the ±10-ms range in the triggered averages of the Ae units (Fig. 17*B*, blue trace), in contrast to the single correlation peak normally created by correlated bias inputs (Fig. 3*B*). The triggered average of the Be units (Fig. 17*B*, red trace) shows the column B spike response to the 10-ms delayed stimulus followed by a second peak produced by the FIC in column B. Further recurrent effects are damped out by unit refractory periods and the inhibitory intracolumn connections (but can become noticeable if the excitatory intracolumn connections significantly outweigh the inhibitory connections). Changes in EPs at different spike-stimulus delays (Fig. 17*D*) are fairly comparable to those in our standard network (Fig. 3*D*); however, many EPs have a secondary peak caused by the FIC in column B (Fig. 17*D*, inset). These secondary effects in column B can be eliminated by removing the FIC in column B, while conditioning effects are still obtained via the FIC in column A.

Similar results were obtained for the other conditioning paradigms tested with FIC networks without correlated bias spikes, namely EMG-triggered, γ-triggered, cycle-triggered, and paired-pulse stimulation. In general, the conditioning effects were somewhat weaker and the tetanic effects (Fig. 17*D*, gray dashed line) were stronger for these FIC networks than for the standard networks. Spike-triggered, EMG-triggered, and γ-triggered stimulation must overcome the more negative than usual effect from tetanic stimulation on column B, while cycle-triggered and paired-pulse stimulation must be compared with the stronger than usual conditioning from tetanic stimulation on column A.

**Figure 18.**
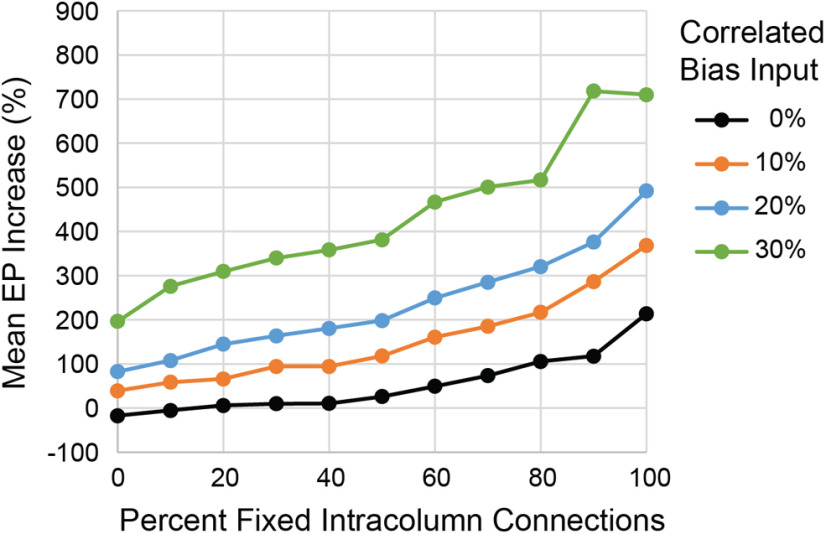
Conditioning effects with different proportions of FICs and correlated external bias input. Curves plot the mean A→B EP increases (averaged over five randomized networks) for spike-triggered stimulation (10-ms spike-stimulus delay).

These conditioning effects can be strengthened by adding varying amounts of correlated bias inputs to the FIC network. Figure 18 shows the conditioned increase in the A→B EP for different proportions of FIC and correlated external input for spike-triggered stimulation. These results suggest that a modified STDP rule for intracolumn connections, one that maintains robust intracolumn weights, could be combined with more modest correlated bias inputs to produce the same conditioning effects as obtained with our standard networks using 30% correlated bias inputs.

## Discussion

Our IF model captures the experimental outcomes from four different conditioning protocols experimentally investigated in motor cortex of awake NHPs. A powerful feature of the model is to explore estimates of many variables that were not measured in the original experiments, such as changes in the connection matrices, sizes of EPs (which measure the synaptic strength of intercolumn connections), and peristimulus histograms of neural activity. Furthermore, the model predicts the outcomes of novel experiments not yet performed.

In comparison, a previous neural network model used populations of Poisson firing units and STDP to analytically compute the population effects produced by spike-triggered stimulation (Lajoie et al., 2017). That model replicated the experimental results on net changes in connectivity and showed that the amount of conditioning was dependent on the correlations between units. That model predicted that conditioning efficacy would be greater when cross-correlation peaks were wider, as they typically are during sleep compared with waking. In contrast, our IF model is based on simulating the synaptic connections between excitatory and inhibitory spiking neurons that integrate synaptic inputs to firing threshold. Our IF network provides cellular resolution of conditioning effects and their dependence on network parameters and simulates many different conditioning protocols.

A similar approach was used to model conditioning with spike-triggered stimulation in somatosensory cortex of NHPs (Song et al., 2013). That study examined the effect of spike-triggered and random microstimulation on firing rates and mutual information in S1. A large network of biophysical units with STDP reproduced many of the global physiological findings. In contrast, our smaller network of much simpler IF units was sufficient to replicate more detailed spatiotemporal measures of conditioning effects.

Other modeling studies have combined IF networks with STDP to investigate related issues. Adding a STDP rule to large populations of conductance-based units (Izhikevich, 2003) led to the emergence of interconnected groups under steady-state conditions (Izhikevich et al., 2004). Our model had fewer units, with predetermined connections between groups, and reached an equilibrium steady state under STDP before conditioning procedures were introduced. Other large-scale cortical network models showed that realistic synaptic plasticity rules coupled with homeostatic mechanisms led to the formation of neuronal assemblies that reflect previously experienced stimuli (Litwin-Kumar and Doiron, 2014) or recall sequences (Klos et al., 2018).

Bono and Clopath investigated STDP and dendritic spike mechanisms in producing plasticity in biophysically realistic neural models (Bono and Clopath, 2017). Another study showed that STDP rules had to be augmented with mechanism for heterosynaptic competition to generate networks capable of producing sequences of neural activity (Fiete et al., 2010). On the behavioral level, a model of orbitofrontal networks of IF units with STDP learned the rules of goal-directed actions (Koene and Hasselmo, 2005). These studies investigated various functional issues, in contrast to our focus on neural mechanisms of synaptic plasticity.

### Differences between the IF model and physiological mechanisms

It seems remarkable that our simple voltage-based IF model could replicate the results of many different physiological conditioning experiments. This robust performance raises the question of where the model has limitations that would inform further refinement. There are several differences between the performance of our model and the original physiological conditioning experiments. First, in the cortical spike-triggered stimulation experiments, not all site pairs developed strengthened connections (Jackson et al., 2006; Rebesco et al., 2010). This may simply be because of a lack of sufficient synaptic connections between those sites to strengthen. However, this explanation would not apply to the original paired stimulation experiments (Seeman et al., 2017), in which many pairs of sites did seem to have sufficient synaptic connections to mediate EPs, but these did not show conditioned changes.

Second, with paired stimulation *in vivo* a minimum of three pulses was required to produce conditioned changes (Rebesco and Miller, 2010; Seeman et al., 2017). In contrast, our IF model exhibited reliable conditioning even with pairs of single pulses, although triplet stimulation was substantially more potent than pairs of single stimuli over a range of intensities. The need for triplet instead of single pulses *in vivo* and the lack of conditioning effects for certain cortical sites remain to be better understood before these physiological observations can inform a change in the model.

A third difference between the model and physiological mechanisms concerns the duration of the conditioned effects. *In vivo*, the duration of effects was related to the amount of conditioning. The briefest conditioning effect (2 s) was produced by triggering stimuli from several cycles of β oscillations (Zanos et al., 2018) and the longest conditioning effect (three weeks) occurred in the behavioral recovery produced by 13 weeks of EMG-triggered spinal stimulation (McPherson et al., 2015). Other protocols involved intermediate durations of conditioning, and these produced correspondingly intermediate durations of effects (Jackson et al., 2006; Lucas and Fetz, 2013; Nishimura et al., 2013; Seeman et al., 2017). In contrast, the time course of the induction and decay of conditioning effects in the model were similar for the different protocols (Fig. 9) and was determined by parameters such as training factor r, shape of STDP function and connectivity.

It seems possible that more sophisticated models, such as a network of biophysically more realistic units would help address some of these differences between the model and physiology. For example, the conductance-based units of Izhikevich capture biophysical properties that can replicate the complex firing properties of many types of cortical neurons in response to prolonged current injection (Izhikevich, 2003; Song et al., 2013). It will be interesting to use the conductance-based units with STDP to simulate the different conditioning protocols investigated here. This will require decisions about the number and connectivity of specific types of units in such a model as well as their biophysical parameters. The fact that our simple voltage-based IF network replicates the overall experimental observations may be related to two factors. It may represent an effective and sufficient average of the different types of biophysical neurons involved in the *in vivo* experiments. Second, the different response properties of biophysical units that can be demonstrated with prolonged intracellular or synaptic drive may be less critical for the phasic events that underly these conditioning protocols.

Our model used a STDP rule that required presynaptic and postsynaptic spikes to generate a change in synaptic connections. Physiologic synapses could also be strengthened when the postsynaptic cell is merely depolarized after arrival of the presynaptic spike (Markram et al., 2012). This would represent a plasticity paradigm that does not require postsynaptic spikes. This mechanism would contribute to conditioning with cycle-triggered stimulation, for example. Intracellular recordings *in vivo* indicate that many cortical neurons show periodic membrane depolarization during β oscillations that do not reach threshold for spiking (Chen, 1993; Fetz et al., 2000). Phase-locked stimulation would modify the strength of synaptic connections to these depolarized neurons as well as to spiking neurons. Our simulations did not track the depolarization level of each unit, so the results of this STDP mechanism remain to be investigated in a future study.

There was a difference between the phase of maximum enhancement for A→B connections for cycle-triggered stimulation: the model showed a maximum at −15° (Fig. 7*B*) and experiments (Zanos et al., 2018) at around −90°. This difference could be due to differences in conduction times, stimulation effects, possible periodicity in activity at other sites, physiological delays and plasticity mechanisms (above) not accounted for in the model.

### Parameter choices

The performance of our IF model depends of course on the choice of parameters. One choice involves the strength of baseline synaptic connections. To capture the small unitary synaptic potentials between cortical neurons (Matsumura et al., 1996; Markram et al., 1997) the plasticity parameters chosen for our model tend to produce a steady-state network with small connections when no activity-dependent conditioning protocol is in effect. This limits the effects produced by tetanic stimulation alone and makes conditioned increases in weights relatively robust. However, it also limits conditioned decreases in weights, since the starting size for most weights is already small. A possible future direction would be to develop a method to encourage a steady state of moderate weights so that both strengthening and weakening effects can manifest more equitably. This might also allow for more effects to emerge from inhibitory units, which play very little role in the network simulations shown here. We found that our standard IF networks that were run without inhibitory units showed conditioning responses similar to networks that had many inhibitory connections.

A second choice in our model was using the same STDP function for inhibitory and excitatory units. The STDP function for inhibitory neurons has been found to vary, depending on the specific types of source and target neurons (Caporale and Dan, 2008; Kullmann and Lamsa, 2011; Vogels et al., 2011; Markram et al., 2012). We decided to use the same STDP function for both, which has empirical support (Haas et al., 2006), and to leave the consequences for different functions for inhibitory units, such as symmetric functions, to be explored in another study. Moreover, our STDP rule is based on pairs of presynaptic and postsynaptic spikes; models with “multispike” STDP interactions can produce different network dynamics (Babadi and Abbott, 2016).

A third choice in our model involved the relative amount of external versus intracolumnar drive on units. Because of the small intracolumn weights the input from local recurrent connections is a small fraction of a unit’s total input, which is dominated by external drive. Thus, each column should be understood as a group of units with similar relations to the recording and stimulation parameters of the conditioning protocols rather than a network of units with sufficient self-connections to sustain substantial dynamics. Other studies have described the network dynamics emerging in populations of more strongly interconnected IF units (Brunel, 2000; Izhikevich et al., 2004; Grytskyy et al., 2013; Wieland et al., 2015; Bos et al., 2016), but the focus of our simulations was on the effects of conditioning protocols on the involved units. Note that the online code for our model does support exploration of different connection strategies.

A fourth choice in our model involved the relative amount of external correlated input bias. High amounts of correlated input can cause sufficient synchrony to strengthen connections from that column even in the absence of conditioning protocols. As the percent of correlated biases increases, the weakening portion of the STDP curve may be increased to prevent this effect from interfering with the manifestation of conditioning effects. For example, in networks with a weakening value of 0.55, using correlated bias ratios above 50% can show more spontaneous conditioning effects because of higher variance in connection strengths over time as compared with networks with our chosen 30% correlated biases.

A related choice in our model was to control intracolumn unit synchrony with external correlated bias. Since intracolumnar weights were reduced by the STDP rule the correlations mediated by intracolumnar connectivity were also reduced. To explicitly investigate the effects of synchrony produced by robust intracolumnar connections we tested networks with strong and FICs and no correlated external bias. These FIC networks also produced conditioning effects for all five conditioning paradigms, albeit somewhat weaker, and showed stronger tetanic effects. Moreover, the FIC produced short-latency secondary peaks in the test EPs (Fig. 17*D*) that generally do not appear *in vivo*. The FIC networks maintained robust intracolumn connections at the cost of artificial inconsistencies in applying the STDP rule to intercolumn but not to intracolumn connections. Similar conditioning results were obtained with FIC and our standard networks, as well as with combinations of FIC and correlated input bias (Fig. 18). Thus, the results obtained with the standard networks manifest the same basic effects, to which stronger intracolumnar connectivity could contribute synergistically.

In general, our choice of network parameters was guided by achieving realistic physiological performance. Notably, the same set of parameters was used to simulate all conditioning protocols. Network performance was generally stable for modest deviations from the chosen parameters. The consequences of larger variations in different parameters would be interesting to explore but are beyond the scope of this report.

### Modeled connectivity and activity

An informative use of the model is to simulate activity and network connectivity that were not measured in the original experiments but that elucidate associated mechanisms. For example, calculating the EPs provides an easy and experimentally feasible measure of net connectivity between sites and can reveal its dependence on conditioning parameters like stimulus amplitude and delay, synchrony, etc. The changes to connection weights themselves can be computed in the model (but not experimentally), these weights closely tracked the EP measure (Fig. 9*A*). The weights further revealed a better time-resolved prediction of the induction and decay of these synaptic connections (Fig. 9*B*).

A particularly useful insight from the model is the activity of relevant units around the stimulus trigger events. Their stimulus-aligned histograms reveal why EMG-triggered and γ-triggered stimulation are such effective conditioning protocols: they both select for coincident unit activity, as shown by the preceding peak in activity of the A units (Figs. 5*B*, 12*B*). This peak preceding the stimulus-evoked postsynaptic activity in B is responsible for the strengthening of the A→B connections.

### Testable predictions

A possible therapeutic application of closed-loop conditioning is to strengthen polysynaptic pathways as well as strengthen direct connections. Such targeted plasticity could restore functional pathways lost to injury or stroke (Guggenmos et al., 2013). The model was used to examine conditioning of disynaptic links by performing A→B paired-pulse stimulation in a network without direct A→B connections and looking for development of A→C→B connections. This did occur, as shown by development of relevant disynaptic EPs after conditioning and by the connectivity matrix (Fig. 14). Similarly, cycle-triggered stimulation could also strengthen disynaptic connections (Fig. 15); however, this protocol would be significantly more challenging to apply *in vivo* than paired stimulation. Both protocols involved sufficient stimulation of A to strengthen A→C connections. The other three protocols involved only stimulation of B and did not strengthen disynaptic A→C→B links. Another significant prediction of the model is that disynaptic effects are conditioned more robustly between sites that lack direct connections as compared with sites that also have direct connections. These simulations provide useful predictions of effective targeted plasticity paradigms and parameters to strengthen polysynaptic pathways.

The model also predicts several conditioning protocols that improve on methods used to date. Instead of spike-triggered single stimuli, the use of spike-triggered bursts of stimuli is more effective, increasing with the number of stimuli (Fig. 10). Second, γ-triggered stimulation is more effective than spike-triggered stimulation because it detects coincident spikes that produce the γ threshold crossing. Importantly, γ-triggered stimulation is experimentally much easier to perform *in vivo* because it does not require long-term isolation of single action potentials. An important caveat here is that the model assumes that γ is generated in a large number of A units that are synchronized and also connected to the B units. The efficacy of γ-triggered stimulation would decrease if fewer units generating the γ LFP in A sent connections to the stimulated units. Preliminary tests of this protocol by R. Yun (unpublished observation) indicate that conditioning effects are less robust *in vivo* in NHP motor cortex than in the model. This indicates that the connectivity of our network, designed to separate the recorded, stimulated and control groups may be too simple to accurately predict experimental outcomes for more complex biological networks in which these functional groups are intermingled with many neurons with other connectivities.

In conclusion, the simple IF spiking network described here has proven remarkably effective in capturing experimental results previously obtained in NHPs with four different conditioning protocols, including three with closed-loop activity-dependent stimulation. In addition to replicating the observed phenomena, the model also allows computation of underlying network behavior and correlated changes not originally documented. The model also makes significant predictions about protocols not yet investigated, including triggering bursts instead of single stimuli and γ-triggered stimulation. The success of these simulations suggests that a simple voltage-based IF model incorporating STDP is sufficient to capture the essential mechanisms that produce targeted plasticity. Further detailed comparisons with physiological experiments will likely inform development of models with more realistic connectivity and biophysical properties.

## Synthesis

Reviewing Editor: Leonard Maler, University of Ottawa Decisions are customarily a result of the Reviewing Editor and the peer reviewers coming together and discussing their recommendations until a consensus is reached. When revisions are invited, a fact-based synthesis statement explaining their decision and outlining what is needed to prepare a revision will be listed below.

Editorial Comments

The remaining reviewer found this Ms to be greatly improved and a potentially important contribution to the field. They do recommend that the nice results re stronger recurrent network connections that the authors be included in the final version. And, some comment should be made re the reviewers note on fixed connections.

These are minor revisions and the next iteration will only need to be reviewed by the Editor.

Please clearly specific in the rebuttal and redlined Ms the changes made.

Reviewer #1

All recommended revisions have been done.

Reviewer #2

The authors have addressed most of my concerns. However, it is a little disappointing that they decided not to include the results obtained from the network with stronger recurrent connections, which they show in the rebuttal.

Global correlations in the columnar activity play a pivotal role in all conditioning protocols, which was already suggested by Lajoie et al. (2017). One potential source of correlations is represented by external input, as in the authors’ model. However, local recurrent connections are also likely to be an important source of correlations in cortical networks, since recurrent connections in the cortex are mostly strong.

I am also not convinced that having some fixed connections in the model necessarily represents an inconsistency, as the authors state in the revised manuscript. Fixed weights in the model could represent synapses with a much slower learning rate, while the assumption that all synapses in the network model follow the same STDP rule with the very same parameters is more of a convenient simplification than a consistency requirement.

I think that incorporating the few extra figures and better discussing this important point would make the study more convincing.

## Author Response

Response to second reviews

We appreciate the consideration of the two reviewers, and have addressed the concerns of reviewer #2.

--

Editorial Comments

The remaining reviewer found this Ms to be greatly improved and a potentially important contribution to the field. They do

recommend that the nice results re stronger recurrent network connections that the authors be included in the final version.

And, some comment should be made re the reviewers note on fixed connections.

These are minor revisions and the next iteration will only need to be reviewed by the Editor.

Please clearly specific in the rebuttal and redlined Ms the changes made.

--

We have added these results to the manuscript, as described below in response to Reviewer

#2

--

Reviewer #1

All recommended revisions have been done.

Reviewer #2

The authors have addressed most of my concerns. However, it is a little disappointing that they decided not to include the

results obtained from the network with stronger recurrent connections, which they show in the rebuttal.

Global correlations in the columnar activity play a pivotal role in all conditioning protocols, which was already suggested by

Lajoie et al. (2017). One potential source of correlations is represented by external input, as in the authors’ model. However,

local recurrent connections are also likely to be an important source of correlations in cortical networks, since recurrent

connections in the cortex are mostly strong.

--

We appreciate this suggestion and have added a section describing and illustrating the

conditioning results obtained with the strong fixed intra-column connections [FIC]. We also

ran additional simulations with the other four conditioning protocols with the FIC network

and found that similar results could be obtained for them as well. These results are

mentioned, but not documented in detail. In addition, we added conditioning results

obtained with various combinations of FIC and external synchronized input. The changes are

highlighted in the reviewer article file.

--

Reviewer #2

I am also not convinced that having some fixed connections in the model necessarily represents an inconsistency,

as the authors state in the revised manuscript. Fixed weights in the model could represent synapses with a much

slower learning rate, while the assumption that all synapses in the network model follow the same STDP rule with

the very same parameters is more of a convenient simplification than a consistency requirement.

--

Agreed. This point is also mentioned in the discussion of the results with the FIC networks.

--

Reviewer #2

2

I think that incorporating the few extra figures and better discussing this important point would make the study

more convincing.

--

We agree and thank the reviewer for emphasizing the importance of addressing the intracolumn connections.

Response to first reviews

We appreciate the helpful feedback from the two reviewers, who clearly understood our study and

made excellent suggestions. We have addressed their concerns as follows.

--

Reviewer #1

The study explores how spike timing dependent plasticity (STDP) can be used in recurrent networks to capture the learning

produced by several different (but related) conditioning protocols.

There are detailed explanations of the simulations and a lengthy exploration of their model. I do have several questions that

should be answered.

1. In Fig 3C there seems to be a slight increase in the B to C and A to C connections. Is there a straightforward disynaptic

understanding of this effect. How would the activity of group C appear in panel B?

--

The increase from B to C is a tetanic conditioning effect caused by repeated stimulation of

column B. The increased A to C connections can be a disynaptic effect. This is sometimes

apparent in the A->C evoked potential (see figure below) which is not shown in Figure 3, but

such a disynaptic effect is shown for the paired triplet stimulation in Figure 16b.

3

Adding the activity of group C to the PSTH in Figure 3B (green trace in figure below) reveals a

response in C to the triggered stimulus on Column B and a very small effect from the

correlated peak in A to C.

We provide this figure to answer the reviewer’s question, but prefer to not replace Figure 3B

with this one because the existing Figure 3B illustrates the essential point and sticks to the

experimentally tested outcomes. Introducing secondary issues about disynaptic effects on

additional columns at this stage in the paper would unnecessarily blunt the main point.

--

2. In comparing Fig 3D and 5D we see that EP increase peaks at a delay of 10ms for the spike triggered stimulations, but shows

no such lag for the EMG triggered stimulation. Yet the response profiles in Figs 3B and 5B do not look very different. Is there a

simple way to understand the difference in Figs 3D and 5D?

--

Most of the apparent discrepancy in delay is accounted for by the 10 ms conduction time

from cortical to motor output units (and a smaller additional delay for the EMG threshold

crossing). Thus, Figure 5D corresponds to the portion of Figure 3D beyond 10 ms. Figure 3B is

aligned on a unit spike trigger so stimulation effects in Column B occur at the 10 ms

conditioning delay. Figure 5B is aligned on the EMG level detection with stimulation effects in

Column B occurring at the same time, with no delay. In both cases, the most effective

stimulation occurs 10 to 13 milliseconds after correlated activity in Column A. The EMG

triggered stimulation just has a longer built-in delay from activity in A due to conduction

times.

To avoid confusion we clarified this by adding in the text: "The delay between these peaks

represents the 10 ms cortico-motor-unit conduction time plus a minor effect of EMG rise to

trigger threshold.". We also modified the caption to read: "(D) The average percent increase

in the EPs evoked in Column B (orange) and C (blue) from stimulating A as function of delay

between EMG threshold crossing and delivered stimulation." and changed the legend for 5D

to trigger-stimulus delay.

4

--

3. The suppressed B to A evoked potentials in Fig. 7D (at 0 degree phase) seem to match the magnitude of the potentials from B

to B and B to C as well as C to A and C to B (see Fig 7C). If the B to A potentials are really suppressed, why are these other

pathways as well?

--

The evoked potentials for B->C, C->A, and A->C do not modulate greatly with the conditioning

phase, as shown in the plots below. They don’t quite reach the level of suppression seen in

the B->A evoked potentials, but approximately match the suppression in A->B at 180 degree

phase. Some of this suppression is due to the STDP weakening curve having larger area than

the strengthening side of the curve. With no tendency towards positive spike time

differences a connection would be suppressed. The figure below is provided to answer the

reviewer’s question but is unnecessarily complicated by extraneous data to include in the

paper.

--

Reviewer #2

The addressed topic and the model are potentially interesting, however, one important limitation needs to be addressed.

Inhibitory neurons have no substantial effect in the proposed model, and there is reason to believe that the same holds for all

recurrent connections (see below). If the recurrent connections of the network barely matter, each cortical column in the

model is essentially equivalent to an ensemble of single neurons with noise. Correlated external noise is needed to synchronize

neurons and make plasticity work, which does not provide new insights into the interplay of STDP and recurrent network

dynamics.

In this computational study, the authors consider a network of integrate-and-fire neurons endowed with STDP. They use this

model to mimic several experiments from the literature, in which different stimulation protocols are used to induce observable

changes in the synaptic strength of the stimulated area. Then, they study the model’s response to some experimentally

untested stimulation protocols.

The paper is well structured and clearly written. The addressed topic is of interest, and a simplified spiking network model is

potentially a useful tool to investigate the dynamics of a recurrent network while avoiding the daunting task of tuning more

biologically detailed models in huge parameter spaces. However, one major limitation renders the model less interesting than it

-100

0

100

200

300

400

500

-180 -90 0 90 180

EP Increase (%)

Conditioning Phase

Col A -> LFP B

Col A -> LFP C

Col B -> LFP A

Tetanic (A -> B)

Col B -> LFP C

Col C -> LFP A

Col C -> LFP B

5

could be.

Major issues:

It is likely that the total recurrent input to each neuron is a small fraction of the total input to each neuron, as the following

estimate suggests.

Each neuron receives 1800 random spikes per second, with a variable degree of correlation. This shot noise models the external

background input to the local cortical column. The number of recurrent input connections from within the network is, on

average, 7 excitatory and 13 inhibitory inputs from within a column, plus 7 additional excitatory inputs from a different column.

Assuming an average firing rate of at most 10 Hz for each neuron (this is estimated from the raster plots as the average firing

rate is an important information missing in the manuscript, while a much higher firing rate would be unphysiological for the

cortex), the number of input spikes/s from within the network is at most 10-15% of the total. Furthermore, the amplitude of

the background input spikes (0.35 mV) is fixed at 70% of the cap for the recurrent connections (0.5 mV), while, after

preconditioning, most recurrent weights are weaker, as stated by the authors on l. 266-267. Hence, the total recurrent input

(considering amplitudes) is an even smaller fraction of the total.

I suspect that recurrent connections barely have any effect in the proposed model. This conjecture is also supported by the fact

that all conditioning protocols need strongly correlated input to work, and that inhibitory neurons make no substantial

difference in the model, as the authors state in the discussion.

When the recurrent coupling of integrate-and-fire networks is not negligible, cross-correlations, oscillations, and slow

fluctuations result from the synaptic interactions between neurons (see e.g. Brunel 2000 J Comput Neurosci 8, Helias et al. 2013

c New J Phys 15, Wieland et al. 2015 PhysRev E 92, Bos et al. 2016 PloS CB 12). In such case, stimulating few or even one single

neuron can have some detectable effect on the network (Martens et al. 2017 J Comput Neurosci 42, Bernardi and Lindner 2017

PhysRevLett 118, van Gils et al. 2019 Neural Comput 31). If, however, the external drive overwhelms the recurrent connections,

the network becomes essentially equivalent to a collection of neurons with shared input plus some uncorrelated noise, so that

the mechanism underlying all conditioning protocols seems to be the synchrony imposed by the external drive.

If the model displayed some intrinsic recurrent dynamics and took into account the effect of inhibition, it would make a much

stronger point and possibly uncover interesting interactions between the STDP rule and the recurrent network dynamics. If the

authors revise their model, they could also take into account the fact that inhibitory neurons in the cortex are much fewer in

number (10 to 20 percent of the total number of neurons), but usually have higher firing rates and stronger synapses.

--

Thank you for pointing out this important issue. When we first started these simulations, it

wasn’t clear how the within-column connections would respond to the STDP rule. Results

showed that our STDP curve tends to suppress connections that are not targeted by

conditioning, and that includes these recurrent in-column connections. We have addressed

this issue in two new paragraphs in the Discussion:

"A third choice in our model involved the relative amount of external vs intracolumnar drive

on units. The input from local recurrent connections is a small fraction of a unit’s total input,

which is dominated by external drive. Thus each "column" should be understood as a group

of units with similar relations to the recording and stimulation parameters of the conditioning

protocols rather than a network of units with sufficient self-connections to sustain substantial

dynamics. Other studies have described the network dynamics emerging in populations of

more strongly interconnected IF units (Brunel 2000, Izhikevich, Gally et al. 2004, Grytskyy,

Tetzlaff et al. 2013, Wieland, Bernardi et al. 2015, Bos, Diesmann et al. 2016) but the focus of

our simulations was on the effects of conditioning protocols on the involved units. Note that

the online code for our model does allow exploration of different connection strategies."

"We found that networks run with many strong and fixed within-column weights could show

conditioning effects with reduced or no correlated bias input. For example, by fully

6

connecting within-column units with connections of 200 to 300 μV and preventing the STDP

rule from modifying them, the various triggered stimulation protocols will cause conditioning

without any external correlated input bias. These networks also show a function for

inhibition to prevent run-away recurrent activity. However, such selective interventions to

artificially boost the intracolumn connections introduce an inconsistency in the network

mechanisms by artificially designating particular fixed connections and are therefore not

included in this report. These simulations can be set up and explored with the online model."

For the reviewer’s benefit, below is a connection matrix and evoked potential graph for such

a network conditioned with spike-triggered stimulation and driven with entirely uncorrelated

bias inputs.

--

Minor issues:

1) The simulation time step of 0.1 ms is only 1/8 of \tau_f, the fast synaptic time scale. This time step seems too coarse,

considering that the authors use a simple Euler scheme. Either a finer time step or a better integration scheme for the faster

variables would be advisable.

--

There is a small difference between the target PSP shape and the shape estimated with

Euler’s method. Originally, we did not include the exponential function and only presented

the Euler scheme, but feedback from other researchers convinced us to include the

exponential function as a familiar point of reference. The estimated PSP shape will conform

closely to an exponential function with slightly different time constants. These time constants

were not meant to be exact but rather representative values.

7

Target PSP Shape Compared to Estimated PSP Shape

We have updated the model to allow for finer time steps. Below are the A->B evoked

potentials from spike triggered stimulation networks run at 0.1 ms timestep and 0.01 ms

timestep showing similar and essentially identical results.

Evoked Potential using 0.1 ms time step Evoked Potential using 0.01 ms time step

To address this issue we have added the option for different time steps in the parameter

table. We also added the following comment on the online code:

"The code includes options not exercised in these simulations, such as including a squashing

function for graded weight changes and running with finer temporal resolution (which did not

significantly alter the main results)."

8

--

2) What is the average firing rate of the network during steady state?

--

When no conditioning is used and the network settles (1000 seconds simulated time), the

base firing rates are mostly set by the bias inputs. There is bit of variation from second to

second, but the cortical units spike between zero and about 15 times in a 1 second time

window. Mean firing frequency is around 7 Hz with a second-to-second standard deviation of

about 2.5 Hz. The output units have graded thresholds and so have an extended distribution

of firing rates between 2 and 25 Hz. Strong conditioning, such as paired pulse stimulation,

can increase the mean firing rates of the targeted units. The following figure shows the

distribution of spike counts inside 1 second windows for units in various column groups using

the last 10 seconds of a simulation run with paired pulse stimulation.

Average unit firing rates for the Ae and Be groups are presently shown in the baselines of the

histograms in Figures 3B, 5B, 6B, 7B, and 12B (which are aligned with conditioning triggers).

This is now clarified in the description for Figure 3B and the captions for the remaining

figures.

"Figure 3B shows the average firing rates of the Ae and Be populations relative to the

triggering Ae1 spike times (T)."

9

--

3) Line 237, and in caption of figs. 8 and 14, the amplitude of the stimulus should be expressed in mV.

--

Thanks for noting this. We fixed this once in Figure 8, twice for Figure 14, and in the text for

the conditioning stimulation and relative efficacy of conditioning paradigms just before Figure

13.

--

4) Is fig. 7D consistent with the experimental outcomes?

--

The experimental outcomes for cycle-triggered stimulation were measured for A→B evoked

potentials; this revealed phase-dependent conditioning (Zanos et al, 2018, Fig. 3C), as was

found with the model (Fig. 7D). The A→C effects were also measured experimentally for

some C sites (Zanos et al, Fig. 5), and showed smaller results that depended on correlations

between oscillations in B and C , as well as variance with the triggered stimuli. When there

were no correlations in vivo, the conditioning effects were negligible. Our model did not

induce oscillations in C, so the A-C effects were relatively weak, consistent with

experimental results. The B-A connections were not experimentally measured but are

included as predictions of the model.

The phase for triggering stimulation that produced maximum enhancement of A -B

connections was about -15 in the model (Fig. 7D). This is somewhat later than with

experimental results showing about -90

(Zanos et al, Fig. 3C) for arrival times of the most

effective volleys. This difference could be due to several different conditions in the model

and the experiments, including conduction times, stimulation and LFP dynamics, and

physiological delays not accounted for in the model. A statement concerning this has been

added to the discussion.

"There was a difference between the phase of maximum enhancement for A-B connections for

cycle-triggered stimulation: the model showed a maximum at -15 (Fig. 7B) and experiments (Zanos et

al, 2018) at around -90. This difference could be due to differences in conduction times, stimulation

and LFP dynamics, and physiological delays and plasticity mechanisms (above) not accounted for in

the model."

--

5) "Testable outcomes": here, the authors investigate effects of the conditioning that are, in their own words, impossible to test

experimentally. Instead of (or in addition to) fig. 9B, it could be studied, for instance, how long does it take for the conditioning

effect to wear off, for a given effect strength.

--

For the network, the amount of time it takes for effects to wear off depends on the balance

between the weakening and strengthing sides of the STDP curve, the firing rates of the units

involved, and the training factor. For the typical values used in these simulations, the effects

wear off within a few minutes of simulated time. We have addressed this point in the results

10

section by including Figure 9C showing the progression of connections strengths from column

A to B over two iterations through the training periods. This figure is described by the added

statement:

"Figure 9C tracks these weight changes during and after two spike-triggered stimulation

periods to show that weight changes can be induced and decay in a minute or two of

simulated time. This figure also shows the variability in conditioning due to the training factor

and overall firing rates of the units. A smaller training factor would reduce the variability of

the conditioned weights but also extend both the training and decay times."

The fact that experimentally the duration of conditioning effects depends on the duration of

conditioning Is addressed in the discussion section by the statement:

"A third difference between the model and physiological mechanisms concerns the duration

of the conditioned effects. In vivo, the duration of effects was related to the amount of

conditioning. The briefest conditioning effect (2 seconds) was produced by triggering stimuli

from several cycles of beta oscillations (Zanos et al, 2018) and the longest conditioning effect

(3 weeks) occurred in the behavioral recovery produced by 13 weeks of EMG-triggered spinal

stimulation (McPherson, Miller et al. 2015). Other protocols involved intermediate durations

of conditioning, and these produced correspondingly intermediate durations of effects

(Jackson, Mavoori et al. 2006, Lucas and Fetz 2013, Nishimura, Perlmutter et al. 2013,

Seeman, Mogen et al. 2017). In contrast, the time course of the induction and decay of

conditioning effects in the model were similar for the different protocols (Fig. 9) and was

determined by parameters such as training factor r, shape of STDP function and connectivity."

--

6) What is the amount of correlated input in the simulations shown in fig. 12?

--

This was our standard 30% of bias spikes are correlated within a column with a Gaussian

function (3ms standard deviation). This has been added to the text as follows.

"Figure 12 shows the effects of stimuli in B triggered from band-passed mid-gamma LFP in

Column A (in 50-80 Hz range, using 30% correlation in the bias spikes)."
